# User engagement in the tuberculosis treatment support tools intervention and its impact on treatment outcomes: A secondary analysis of a pragmatic trial

**DOI:** 10.1371/journal.pdig.0001457

**Published:** 2026-07-02

**Authors:** Sarah J. Iribarren, Jason Rupp, Jennifer Sprecher, Barry Lutz, Fernando Rubinstein

**Affiliations:** 1 Biobehavioral Nursing and Health Informatics, University of Washington, Seattle, Washington, United States of America; 2 Department of Bioengineering, University of Washington, Seattle, Washington, United States of America; 3 Institute for Clinical Effectiveness and Health Policy (IECS), Buenos Aires, Argentina; University of Vic - Central University of Catalonia, SPAIN

## Abstract

Digital adherence technologies (DATs) may improve health behaviors only when users engage, but links between engagement, user factors, and outcomes are unclear. TB Treatment Support Tools (TB-TST) is a DAT with a smartphone app connecting patients to treatment supporters and weekly drug metabolite testing. We evaluated TB-TST app engagement in a pragmatic randomized controlled trial and identified factors associated with adherence and treatment outcomes. Engagement was measured from app interactions by 277 participants over 180-days of TB treatment. Adherence was assessed via daily self-reports and weekly metabolite-test photo submissions. Participants could also message treatment supporters, report side effects, or request help. We modeled time to non-adherence (28 consecutive days without reporting) using survival analysis. Logistic regression tested associations of adherence and engagement with treatment outcomes. A latent engagement score was derived using confirmatory factor analysis (CFA) from medication reports, photo submissions, side-effect reports, messaging, and overall adherence. Participants submitted 24,902 medication reports, 2,926 messages, 2,465 photos, 1235 side effect reports, and 128 help requests. Adherence declined over time (78% at 60 days; 50% at 180 days). Non-adherence was more common among males, participants living at or below the poverty line, those without stable employment, and those treated at certain hospitals. Non-adherence was associated with lower odds of treatment success (OR 0.48, 95% CI: 0.22 - 0.94) and higher odds of loss to follow-up (OR 2.1, 95% CI: 1.03 - 4.7), adjusting for sex, age, education, income, and employment. Higher engagement scores were associated with higher odds of success (OR 2.2 times per standard deviation increase). Engagement with TB-TST was associated with improved TB treatment outcomes. Strategies to increase and sustain engagement, particularly among high-risk groups, may improve adherence and maximize DAT benefits.

## Author summary

Tuberculosis treatment lasts for many months, and interruption or incomplete therapy can lead to drug resistance and other poor outcomes. We studied how people used a smartphone-based support tool during tuberculosis self-administered treatment and whether more consistent use was linked to better outcomes. Digital tools can help patients take medications regularly, but they only work if people continue to use them over time. In our trial, the tool allowed participants to report when they took their daily dose, share a weekly photo of a simple test that indicates recent medication use, and communicate with a treatment supporter when they had questions, side effects, or needed help. We found that engagement with the tool decreased as treatment went on. People were more likely to stop reporting for long periods if they were men, had low income, lacked stable employment, or received care at non-referral hospitals. Importantly, people who stopped reporting were less likely to complete treatment successfully and more likely to be lost to follow-up. In contrast, people who used the tool more consistently had better treatment outcomes. These results suggest that digital support can strengthen tuberculosis care, but programs should invest in practical ways to keep people engaged, especially among groups at higher risk of falling behind.

## Introduction

The expansion of Digital Adherence Technologies (DATs) offers promising strategies for cost-effective, patient-centered solutions to address challenges of complex tuberculosis (TB) treatment [1]. TB remains the leading infectious killer globally and contributes significantly to morbidity and mortality [2]. Its treatment involves lengthy antibiotic regimens with potential side effects, which creates substantial barriers to patient adherence. Poor adherence compromises individual health and exacerbates public health concerns by fostering drug-resistant TB strains and increasing disease transmission [3].

DATs designed for TB management have predominantly focused on video-based monitoring and smart pill bottles, while fewer studies have evaluated interactive mobile applications (apps) [4]. Mobile apps hold the potential to enhance personalized treatment supervision, empower patients in self-management, and improve patient-provider communication through advanced functionalities like real-time support and monitoring [5]. However, most existing TB apps target healthcare providers (e.g., dose calculations) or offer general TB information, rather than actively engaging patients in their care [6]. Few apps incorporate self-tracking features, side effect monitoring, or objective adherence measures like drug metabolite tests [6,7]. Moreover, none have been tailored specifically for people of Latin American decent or Spanish-speaking populations, despite the Americas region having one of the lowest TB treatment success rates globally and a high proportion of Spanish speakers affected by the disease [8]. Existing reviews of TB-related digital interventions report mixed effectiveness, which underscores the need to examine factors influencing their impact [9].

The success of DATs in improving health outcomes largely hinges on user engagement, which is often measured as frequency of use [10]. Engagement is crucial for overcoming barriers, such as time constraints, travel difficulties, costs, and stigma, while reducing the burden on healthcare systems. Despite its importance, engagement remains underexplored. App-based interventions are often evaluated using traditional methods like intent-to-treat analysis, which fail to capture the nuanced relationships between engagement patterns and health outcomes [11]. Understanding how users interact with app features and how these interactions impact treatment outcomes is critical for refining digital interventions, identifying areas for improvement, and supporting users who may require additional engagement strategies.

**Fig 1:**
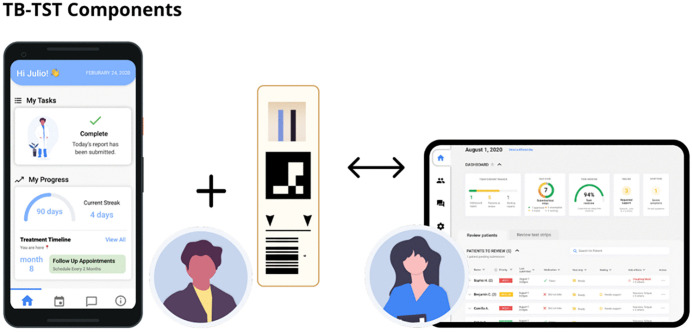
Tuberculosis treatment support tools components.

This study aims to enhance the design and effectiveness of digital health interventions by addressing key research gaps related to user engagement and its impact on treatment outcomes. Specifically, it evaluates participant engagement with the TB Treatment Support Tools (TB-TST) intervention – a DAT featuring a comprehensive app that connects patients with treatment supporters and incorporates an objective adherence test (Fig 1). Using data from a pragmatic, multi-site clinical trial, the study explores associations between user characteristics, app usage patterns, and treatment outcomes.

The TB-TST intervention was designed in collaboration with patients and experts in Argentina, a country with historically low TB treatment success rates (44–67%), high rates of loss to follow-up, and a high reliance on self-administered treatment practices [12]. The incidence of TB in Argentina has increased by 37% since 2015 [13]. By examining how engagement patterns relate to treatment outcomes, this research provides actionable insights to guide the development of more effective DATs, with the goal of enhancing patient engagement, improving adherence, and ultimately supporting TB treatment success, particularly in high-burden, resource-limited settings.

## Methods

### Overview

This study is a secondary analysis of a parallel-group, pragmatic randomized controlled trial (RCT) that evaluated the efficacy of the TB-TST intervention in improving TB treatment success compared to standard care. This secondary analysis focuses on the intervention arm, which examines participant app usage and its association with treatment outcomes. Detailed information on the primary trial study design, procedures, and primary findings is reported elsewhere and described in brief below [14]. The analysis of level of engagement was planned *a priori* in the original trial protocol, and confirmatory factor analysis (CFA) was used to derive the engagement construct based on its theoretical and methodological strengths in validating its relationship with the proposed observed variables [15]. Engagement with DATs is a multidimensional construct that may include behavioral (e.g., app usage frequency), cognitive (e.g., perception of usefulness), and emotional (e.g., motivation or satisfaction) components. CFA enables testing whether observed indicators (e.g., self-reported items, app interaction metrics) reliably load onto a predefined latent engagement factor that is grounded in theory or prior empirical work [16,17]. CFA, unlike exploratory factor analysis, enables hypothesis-driven testing of a predefined measurement model. This is particularly relevant in the context of intervention evaluations, where engagement is conceptualized beforehand and operationalized through specific, observable behaviors and responses.

The objectives of this analysis were to: (1) describe app usage patterns, (2) identify participant characteristics associated with adherence to the intervention, and (3) define and analyze the complex concept of engagement by exploring its relationship with treatment outcomes. We hypothesized that higher engagement with the app would be significantly associated with treatment success. To achieve these aims, engagement was operationalized as a multidimensional construct and included metrics of frequency of medication intake reports, photo submissions of drug metabolite urine strip tests, reporting side effects, direct communication with treatment supporters, and overall adherence.

### Ethical approval

The primary trial was registered at ClinicalTrials.gov (NCT 04221789). Study procedures were approved by the Institutional Review Boards of the Ministry of Health of the Province of Buenos Aires (ACTA-2019–15552860-GDEBA-CECMSALGP) and the University of Washington (STUDY00007533). All participants provided written informed consent. Each participant was assigned a unique study identification at screening, and the linkage file was maintained securely within REDCap. For analysis, direct patient identifiers were removed, and only study identification numbers were used. Data were stored in approved secure folders accessible only through authenticated, password-protected logins.

### Study procedures

The data used for these analyses were from baseline, 6-month (180 day) of TB treatment, and final treatment outcomes. Eligibility criteria were: (1) newly diagnosed with drug-susceptible TB, (2) aged ≥16 years, and (3) having regular access to a smartphone and being able to operate it independently or with assistance. Exclusion criteria included presenting with severe illness requiring hospitalization, confirmed drug resistance, and cohabiting with another participant. Participants were recruited in-person from four large public health hospitals in Argentina on a rolling basis as they were diagnosed with TB from November 2020 through December 2022, 6-months usage was completed in July 2023, and final outcomes measures were received in November 2023.

Eligible participants underwent informed consent procedures before enrolling in the study. After enrollment, participants completed an in-person baseline survey and were randomized (in 1:1 ratio) to either the control group or the intervention group. The control group received standard TB care, and the intervention group received standard plus the TB-TST intervention during the standard 6-month treatment course.

### Onboarding and training

Onboarding was provided in person by trained personnel who assisted participants with installing the app and completing a test submission. The onboarding session included a brief orientation to app navigation, the medication reporting process, and test photo submission supported by short in-app tutorials. Participants received verbal instructions as well as print, in-app and video materials.

### Intervention

**Fig 2:**
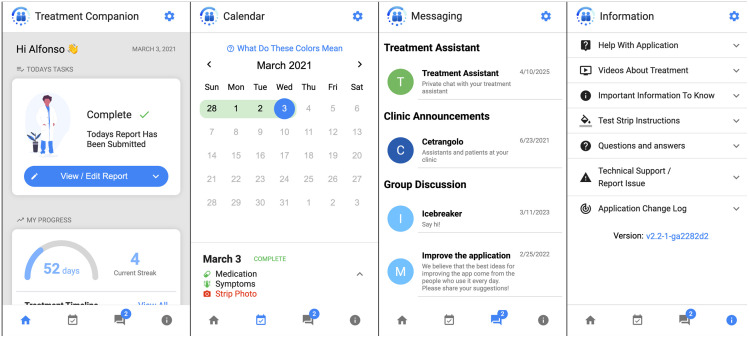
Screenshots of main TB-TST app features.

The TB-TST intervention was iteratively developed with clinical and human-center design expertise in collaboration with TB experts and individuals with TB, and it was grounded in the Information-Motivation-Behavioral Skills Model (IMB) and related behavior change techniques [18–20]. App features designed to support patients in maintaining treatment adherence remotely through their smartphone included: (1) TB education (e.g., written and video information about TB and its treatment), (2) daily survey to submit reports of medication self-administration, side effects, or request of help, or a one-step option to confirm medication was taken and no issues to report, (3) treatment progress visualization (e.g., reporting streak, days in treatment, upcoming appointments), (4) message channels (allowing direct communication with a treatment supporter or participation in an anonymous group forum with other app users), and (5) test instructions and photo submission. Fig 2 illustrates four of these app features. The intervention is reported using the TIDieR (Template for Intervention Description and Replication) Checklist (S1 Checklist).

The intervention was delivered in three public hospitals in the Province of Buenos Aires, with participants using the app at home on personal smartphones. Once per week, on a randomly selected day, the app sent a push notification requesting participants to conduct a drug metabolite test and upload a photo of the results. The test is a colorimetric assay detecting isoniazid (INH) metabolites in the urine by turning blue/purple if INH was ingested within the past 24 hours [21,22]. Sensitivity and specificity are high (97% and 98% respectively) when the test is used within 24–30 hours of ingestion [23–26]. Because INH is a key component of many fixed-dose TB treatments, the test provides a reliable indicator of recent adherence. Participants uploaded a photo of the test strip to confirm adherence.

Additionally, participants received weekly push notifications with information, tips, encouragement, behavior-change strategies, and reminders tailored to their treatment phase. Participants had access to the app throughout their 6-month treatment period. Daily surveys could be submitted once per day, and weekly notifications and test requests were automatically scheduled by the system. Tailoring occurred at the notification level, with automated messages adjusted based on treatment phase (intensive vs. continuation) and prior engagement patterns.

### Treatment supporter role and fidelity monitoring

Treatment supporters (physicians and social workers) reviewed the dashboard daily during clinical hours to assess participant activity, respond through secure messaging, and review and code test trip images. They received standardized instruction on how to interpret dashboard data, respond to participant requests, and apply behavior-change communication strategies within the app’s messaging platform.. Fidelity monitoring was conducted through monthly reports summarizing engagement and adherence, which were sent to treatment supporters. All app data were stored securely and in compliance with HIPAA standards.

### Measures

This study used baseline demographic data, app usage metrics (during the 180-day intervention period), and treatment outcomes obtained from medical records.

*Demographics*. Baseline variables included age, sex, education, nationality, marital status, employment status, and household income. Income was measured by asking participants to select their monthly income range.

*App use*. Metrics included daily reports of medication intake, side effects, test photo submissions, help requests, and messaging activity.

*Adherence to daily medication reporting*. Reporting adherence was defined as the number of consecutive days with medication intake reporting. Non-adherence was operationalized as 28 or more consecutive days of non-reporting. We selected 28 days a priori to align with monthly TB medication dispensing cycles and routine monthly follow-up in the study setting, which provided a programmatically meaningful threshold for sustained disengagement while limiting misclassification from short, transient gaps (e.g., connectivity issues or acute illness). We conducted a 14-day cutoff sensitivity analyses, and adherence measures were standardized to the 180-day period.

*Engagement*. Engagement was conceptualized as a latent variable derived from app use metrics via CFA. These included daily reports of medication intake, side effect reports, test photo submissions, number of messages, and overall adherence.

*Treatment outcome*. Treatment outcome was extracted from medical records and classified per WHO definitions: treatment success (cured or completed), treatment failure, loss to follow-up, death, or not evaluated [27]. Patients who deviated from the study protocol (e.g., diagnosed with drug-resistant TB post-enrollment, extended hospitalization) were assessed to determine whether they were loss to follow-up before the date of protocol deviation.

### Analysis

All analyses were conducted using Python 3.9.12, STATA 18 and R v 4.3.3. Participants were included if they registered in the app and submitted at least one post-training report. Descriptive statistics (means, SD, frequencies) were calculated for baseline variables. The significance level was set at α = 0.05. Chi-square tests assessed differences in adherence by demographic characteristics. The dataset used for demographic analysis contained no missing or invalid data.

Survival analysis assessed non-adherence, similar to Edney et al. 2019 [28], using Kaplan-Meier curves and log-rank tests. Cox regression and Fine and Gray models were used to examine predictors of non-adherence, with covariates including age, sex, education, employment, and recruitment site. The time variable was the number of app usage days for self-reported medication intake. Observations were censored at the end of the 180-day period or treated as a competing event if lost to-follow-up.

Confirmatory Factor Analysis (CFA) was used to evaluate the factor structure of a set of observed variables (app use metrics included daily reports of medication intake, side effects, number of strip test photo submissions, number of messages, and overall adherence) and to test the hypothesis that a relationship exists between those observed variables and the underlying latent construct of *Engagement* with the app. We used factor loadings (pattern matrix) and unique variances for each variable to compute a standardized factor score (mean = 0, SD = 1) that represents a level of *Engagement* with the app for each patient. That value was then entered into logistic regression models to examine its association with treatment outcomes. Since the score is standardized, the coefficient represents the expected difference in outcome associated with a unit change (one SD) in the engagement score.

## Results

### Participant demographics

Participant demographics, stratified by adherence to daily app reporting, are presented in Table 1. The sample was nearly balanced by sex (female 144, 52%), with a mean age of 33.7 years (SD 13.61; range 16–79). Most participants reported completing at least primary school education (261, 94%). Approximately half of the participants were employed at the initiation of treatment (143, 51.6%), and fewer than half (119, 42.9%) reported having a spouse or partner. Most participants were Argentinean (213, 76.9%), and nearly half reported household income at or below the poverty line (127, 45.8%).

**Table 1:** Participant sociodemographic characteristics by adherence to daily reporting.

		Adherent	Nonadherent	*p*
n		130	122	
Sex, n (%)	Female	75 (58.1)	59 (48.5)	0.108
	Male	54 (41.9)	63 (51.6)	
Age, mean (SD)		32.6 (11.5)	33.8 (14.8)	0.468
Level of Education, n (%)	Primary	25 (19.2)	31 (25.6)	0.118
	Secondary	77 (59.2)	75 (62)	
	Post-Secondary	28 (21.5)	15 (12.4)	
Steady Employment, n (%)	Yes	76 (58.5)	55 (45.1)	**0.034**
Income Level, n (%)	At or below the Poverty line	80 (66.1)	79 (72.5)	0.227
	Above the Poverty line	41 (34)	30 (27.5)	
	No Answer	9 (7)	13 (10.0)	
Marital Status, n (%)	Married/ Stable Relationship	71 (54.6)	68 (55.7)	0.10
Argentine National, n (%)	Yes	100 (76.9)	93 (76.2)	0.897

We assessed potential differences in participant demographics based on adherence to daily app reporting. No significant differences were observed between adherent and nonadherent groups across socio-demographic characteristics, except for steady employment.

Participants submitted 23,103 reports of medication intake, averaging 91.7 reports per participant (SD 65.6; range 1–180), 2,926 messages, averaging 11.5 per participant (SD 19.9; range 0–147), 2,343 photo submissions averaging 9.3 per participant (SD 11.4; range 0–66), 1,235 reports of side effects averaging 4.9 per participant (SD 13; range 0–87), and 128 requests for help averaging 0.5 per participant (SD 2.1; range 0–27).

### Reporting patterns

**Fig 3:**
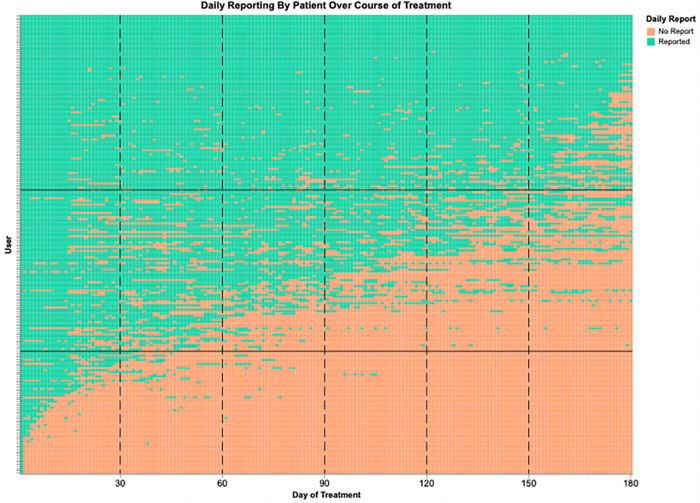
Heatmap of daily medication reporting. Each horizonal row indicates daily reporting for one participant over 180-days of treatment. Green box indicates a medication intake report that was submitted that day, and orange indicates a missing report. Horizontal black lines indicate cutoff point of >80% reports (top) and <20% (bottom).

The frequency of daily medication reporting varied among participants, as illustrated in the daily medication intake reporting heatmap (Fig 3).

### Side effects reported

Of the 1,235 side effect reports, the three most common issues were gastrointestinal-related: abdominal pain, nausea, and appetite loss. Other frequently reported side effects included rash, fever, difficulty breathing, blurry vision, and hives.

### Treatment outcomes

**Fig 4:**
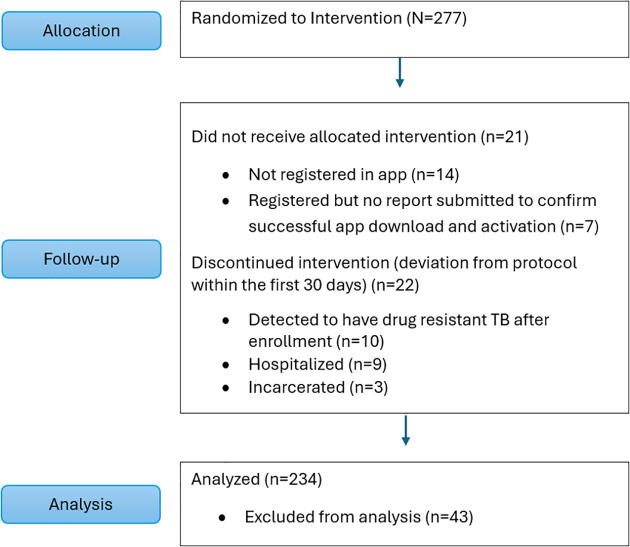
Analysis flow diagram.

Of the 277 participants randomized to the intervention, 234 were included in the final outcome analysis (Fig 4. Analysis Flow Diagram). Participants were excluded if they were not registered in the app (n = 14), registered but did not submit at least one report to confirm successful app download and activation (n = 7), or deviated from protocol within the first 30 days (n = 22). Among the 234 participants included, the majority achieved treatment success (197, 84.2%), while 37 participants (15.8%) experienced poor treatment outcomes (Table 2). Among those who did not receive the allocated intervention and were excluded from the analysis (n = 21), 10 completed treatment, 1 was cured, and 10 were lost to follow-up.

**Table 2:** Treatment outcomes among participants included in analysis.

Outcome	N = 234 (%)
Completed Treatment	156 (66.7)
Cured	41 (17.5)
Loss to Follow-up	34 (14.5)
Died	2 (0.008)
Failure	1 (0.004)

### Adherence on treatment outcomes

Logistic regression analysis demonstrated that 28-day non-reporting adherence was significantly associated with poor outcomes. Non-reporting adherence had lower odds of success (OR 0.48, 95% CI: 0.22 - 0.94) and higher odds of lost to follow-up (OR 2.1, 95% CI: 1.03 - 4.7) when adjusting for sex, age, education, income, and employment status.

### Factors associated with non-reporting (survival analysis)

**Fig 5:**
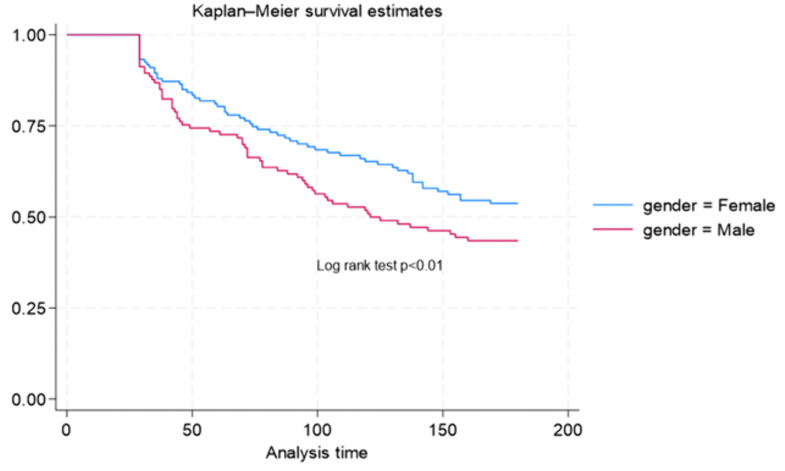
Survival estimates time to 28-day non-reporting adherence by gender.

Survival analysis identified three significant predictors of 28-day non-reporting: being male, having an income at or below the poverty line, and being unemployed (Table 3). Daily reporting declined over time, with 78% of participants adherent at 60 days and 50% at 180 days (Fig 5).

**Table 3:** Proportional hazard test based on Schoenfeld residuals.

Time function:	Analysis time			
	rho	chi2	df	*P* value
Male Sex	-0.03823	0.16	1	0.6936
Income	.	.	1	.
Poverty	-0.08545	0.82	1	0.3665
Above poverty	-0.04585	0.24	1	0.621
Stable employment	-0.02857	0.09	1	0.7645
TB Reference Hospital 1	-0.0243	0.07	1	0.7959
**Global test**		**1.29**	**5**	**0.9355**

The proportional hazard assumption was met; Schoenfeld residuals indicated no significant departures globally or for individual covariates (Table 3). Cox proportional hazards model and Fine-Gray competing risk models produced nearly identical estimates, likely because only 11 participants experienced the competing event (loss to follow-up) before reaching the main event (28-day non-reporting) (Table 4 and S1 Fig).

**Table 4:** Cox regression multivariable and fine-gray models: Time to 28-day non-reporting adherence.

	Cox Regression Multivariable Model		Fine-Gray Multivariable Model	
	HR (95% CI)	*P* Value	HR (95% CI)	*P* Value
Male Sex	1.79 (1.2 - 2.6)	0.004	1.75 (1.186 - 2.59)	0.005
Income				
Below poverty line	1		1	
At poverty line	0.54 (0.33 - 0.89)	0.015	0.56 (0.36 – 0.87)	0.011
Above poverty line	0.47 (0.27 - 0.81)	0.007	0.48 (0.29 – 0.80)	0.005
Stable employment	0.61 (0.41 - 0.92)	0.018	0.59 (0.40 – 0.89)	0.012
TB Referral Hospital 1	0.42 (0.28 - 0.63)	<0.001	0.44 (0.30 – 0.65)	<0.001

CI indicates confidence interval: HR, hazard ratio.

The Cox model estimates hazard ratios (HRs) for the time to non-reporting adherence, treating loss to follow-up as a non-informative censored event. The Fine-Gray model estimates sub-distribution hazard ratios (SHRs) accounting for loss to follow-up and describing effects on the cumulative incidence of non-reporting adherence while retaining those who experience the competing event in the risk set.

### Interpretation of gender and employment effects

Across both models, males had a 75–79% higher risk of non-reporting adherence compared with females. Adjusting for age did not alter the association; age was neither a confounder nor an effect modifier. Stable employment was associated with a 38–41% lower risk of non-reporting adherence which indicates that employment stability is an independent protective factor. These effects remained statistically significant and clinically meaningful after adjusting for other model variables. The similarity between HR and SHR estimates suggests that the competing event had minimal effect on the effect sizes. Results were consistent in the sensitivity analysis using 14-day non-reporting adherence (S1 and S2 Tables).

### Engagement and treatment outcome (confirmatory factor analysis)

We used confirmatory factor analysis (CFA) to test a unidimensional engagement construct based on four observed indicators; (1) daily reports of medication intake, (2) number of messages, (3) number of photo submissions, and (4) 28-day non-reporting adherence (S2 Fig. Data suitability checks supported factor modeling; the Bartlett test of sphericity was significant (p < 0.001), and the Kaiser–Meyer–Olkin measure of sampling adequacy was 0.68, which indicated acceptable sampling adequacy.

An exploratory check identified a single factor (eigenvalue of 2.49) explaining 62% of total variance, which is consistent with a unidimensional structure. CFA fit indices indicated excellent model fit: Comparative Fit Index (CFI) and Tucker-Lewis Index (TLI) = 0.99 (above the 0.95 benchmark), and Standardized Root Mean Squared Residual (SRMR) = 0.026, which suggests minimal residual discrepancy. The Coefficient of Determination (CD) = 0.835 suggests that the model explains ~84% of the variance (S3 Table).

Standardized factor loadings (Table 5) were uniformly positive and substantively meaningful. Using regression scoring, we computed a standardized engagement score as a weighted sum of standardized indicators with weights 0.36, 0.22, 0.32 and 0.34, respectively (Table 6).

**Table 5:** Pattern matrix (Factor loadings).

Variable (Observed Indicator)	Factor Loading	Unique Variance
Daily medication reports	0.91	0.18
Messages sent/received	0.57	0.68
Photo submissions	0.79	0.37
Adherence	0.85	0.28

**Table 6:** Weights of app engagement variables.

Variable (Observed Indicator)	Weight
Daily medication reports	0.36
Messages sent/received	0.23
Photo submissions	0.32
Adherence	0.34

The standardized engagement score (mean = 0, SD = 1) was included as a predictor in logistic regression models to examine its association with treatment outcomes. After adjusting for sex, age, education, income, and employment status, higher engagement was significantly associated with improved outcomes. For each one-standard deviation increase in engagement, the odds of treatment success increased (OR 2.2, 95% CI: 1.36-2.53, p < 0.001), while the odds of loss to follow-up decreased (OR 0.43, 95% CI: 0.26-0.72, p < 0.001).

## Discussion

### Principal findings

This study examined how engagement with a DAT relates to TB treatment outcomes. Higher engagement with the TB-TST app was associated with significantly greater odds of treatment success, whereas declining engagement, particularly non-adherence to daily reporting, was linked to poorer outcomes and higher loss to follow-up. Consistent with patterns documented across many digital health interventions [29], we observed a steady decline in engagement over time, which highlights the need for strategies to sustain participant use throughout the treatment period. A novel contribution of this study is the use of confirmatory factor analysis to conceptualize engagement as a multidimensional underlying construct, which offers a more integrated and theory-driven framework for measuring and improving DAT performance.

We also found that women demonstrated higher DAT adherence after adjusting for potential confounders. This result aligns with broader digital health research showing that women often engage more consistently with mobile health tools [30]. Although age was neither a confounder nor an effect modifier of the gender-engagement relationship in our study, prior research has identified older age as a risk factor for lower engagement [31,32]. These patterns suggest the importance of tailoring DATs to better support men and older adults, with the goal of promoting more equitable engagement and adherence.)

### App use patterns

App use declined from 78% at 60 days to 50% at 180 days, which mirrors trends observed in other mobile health interventions [28,33]. Some of this decline may reflect habit formation, as participants reported increase confidence in their treatment routines and reduced reliance on reminders. For those experiencing fewer side effects or those with stronger support systems, the perceived value of daily reporting may have diminished over time. Connectivity issues were also reported, which had been anticipated during app development. To address this, the app was designed with offline functionality, which allowed participants to enter data without internet access, with automatic upload once a connection was restored. This likely contributed to the observed pattern of intermittent reporting. Treatment supporters assisted with app-related connectivity or access issues during clinic visits. To mitigate drop off in engagement, future iterations could explore phase-based or intermittent reporting schedules that reduce user burden while maintaining effective treatment monitoring. Optional or adaptive features tailored to users’ needs may further support engagement over time.

### Gender and engagement disparities

Male sex was significantly associated with higher rates of non-adherence, consistent with literature on gender disparities in TB outcomes and some research on digital health tool uptake [34,35]. Possible explanations include lower perceived benefit, greater work-related constraints, and masculine norms that devalue help-seeking behavior. Prior studies have shown women often engage more consistently with mHealth tools, potentially due to higher health literacy, greater comfort with health communication, or stronger social motivation [36]. Designing gender-sensitive features, such as flexible check-in options, alternative onboarding formats, or targeted motivational messages, may help address these disparities and support improved outcomes among men [37].

### Multidimensional engagement and outcomes

User engagement with digital behavior change interventions is considered important for their effectiveness, therefore, evaluating engagement is important [15]. Using CFA, we identified a unidimensional engagement construct that integrated app metrics (daily medication reporting, messaging, test photo submissions, and adherence). This composite score was significantly associated with better treatment outcomes. Unlike analyses that assess app features in isolation [28,38], our approach accounts for the interplay among behaviors and reflects more realistic usage patterns. Engagement also varied by individual need. For some, messaging with the treatment supporter was the most valued feature. However, the effectiveness of such interactions likely depends on treatment supporter empathy, responsiveness, and communication quality. Maintaining consistency and scalability in personalized digital support remains a challenge for provider-augmented interventions.

### Onboarding and early use challenges

A notable number of participants failed to onboard or sustain use beyond the few days. The COVID-19 pandemic disrupted in-person support and limited follow-up, especially for participants who did not have their phones at enrollment. Additional possible barriers include male gender, older age, and limited digital literacy, which are factors also highlighted in prior research on mHealth adoption. Addressing these challenges will require more robust onboarding strategies, including additional follow-up support after enrollment, simplified app interfaces, clearly communicate of purpose and benefits, and differentiated support for users with limited digital familiarity.

### Strengths and limitations

Strengths of this study include large sample, a 6-month longitudinal follow-up period, and the development of a composite engagement score that reflects the interrelated nature of app features. Our operationalization of non-adherence (e.g., ≥ 28 consecutive missed days) allowed us to distinguish between sporadic and prolonged disengagement, which offered a more nuanced view than aggregated frequency measures.

This study has several limitations. First, the secondary observational design precludes causal inference, and unmeasured confounding may have influenced patterns of engagement. Selection bias is possible, as individuals who consent to participate may have been more motivated, more comfortable using smartphones, or had greater digital literacy than those who declined enrollment. Pandemic-related constraints during onboarding that led to high numbers of randomized participants not downloading the app and receiving the intervention, such as limited in-person training, forgetting phone, unable to return for app download assistance, and varying levels of hospital staffing, may have further contributed to differences in initial familiarity or initiating the intervention. Finally, generalizability may be limited. The trial was conducted in public hospitals within the Argentine health system, where patterns of care, patient demographics, cultural norms, and smartphone use may differ from those in private-sector settings or in other countries. Although the mechanisms underlying app engagement may be relevant to similar resource-constrained environments, findings should be interpreted with caution when applied to health systems with different digital infrastructure, patient support mechanisms, or care pathways.

### Implications for digital health interventions

Our findings highlight the need to tailor digital health tools to users’ preferences, routines, and engagement patterns, particularly for conditions requiring long-term treatment, such as TB. Sustained engagement may require phase-based or adaptive strategies that reduce burden while maintaining clinical oversight. Robust onboarding support and gender- and age-sensitive design may improve uptake and retention. Lastly, standardized frameworks for conceptualizing engagement, such as factor-based scoring, can strengthen future evaluations and help optimize digital tools for diverse populations. As digital interventions become more central in global health, especially resource constrained settings, understanding how and why users engage, or disengage, will be essential to their success.

## Conclusion

This study highlights the critical role of app engagement in supporting successful TB treatment outcomes. Findings point to the need for refining the TB-TST intervention to overcome onboarding barriers and sustain engagement throughout the treatment course. Future research should focus on strategies to optimize user interactions, reduce reporting burden, ensure consistent treatment support, and tailor digital tools to diverse patient needs. The engagement score developed here offers a promising metric for use as a stratification variable or mediator in future evaluations. By addressing these challenges, DATs can more effectively promote treatment adherence and improve health outcomes in resource-limited, high-burden settings.

## Supporting information

S1 Checklist TIDieR-Checklist. (DOCX)

S1 Fig Supplementary analysis: Forest plot predictors of non-adherence. (DOCX)

S2 Fig Supplementary analysis: Structural equation model of engagement. (DOCX)

S1 Table Supplementary analyses: Non-adherence according to 14-day and 28-day cut off definitions. (DOCX)

S2 Table Supplementary analysis: Cox proportional hazards models for predictors of non-adherence at 14 and 28 days. (DOCX)

S3 Table Supplementary analysis: Goodness-of-fit indices for the structural equation model. (DOCX)
