# Supplementary figures and images for: User engagement in the tuberculosis treatment support tools intervention and its impact on treatment outcomes: A secondary analysis of a pragmatic trial
Source: PLOS Digit Health. 2026 Jul 2;5(7):e0001457. doi: 10.1371/journal.pdig.0001457 (PMC13327242; doi:10.1371/journal.pdig.0001457)

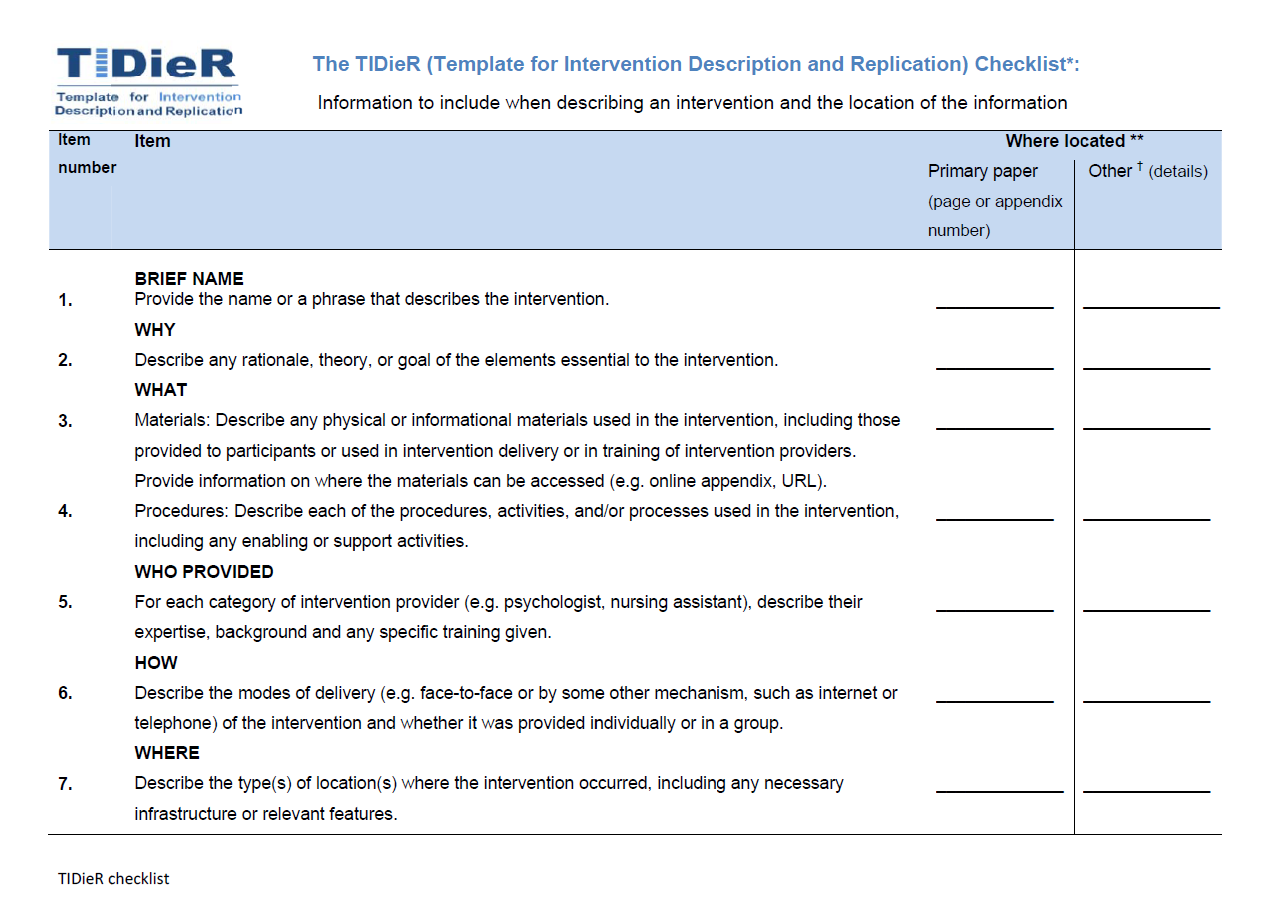


9, lines 141-142, 158-159

9, lines 158 159

9, lines 176-178

9, lines 158-178

9, lines 156-159

1

8, lines 151-153


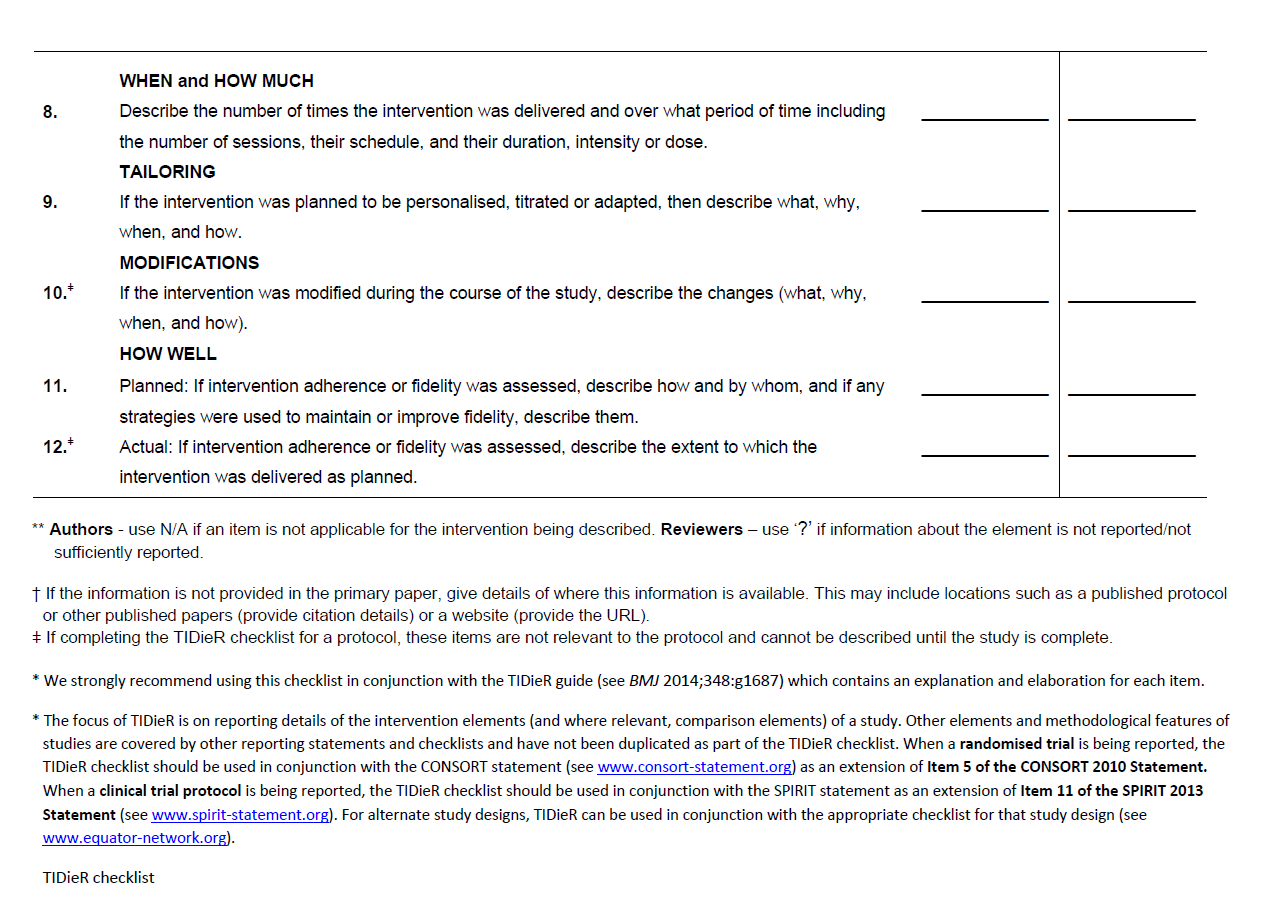


This paper focuses on this

10, lines 184-187

10, lines 181-183

10, lines 179-181

Supplement: S1 Checklist — (DOCX) [file pdig.0001457.s001.docx]
