# Supplementary material for: User engagement in the tuberculosis treatment support tools intervention and its impact on treatment outcomes: A secondary analysis of a pragmatic trial
Source: PLOS Digit Health. 2026 Jul 2;5(7):e0001457. doi: 10.1371/journal.pdig.0001457 (PMC13327242; doi:10.1371/journal.pdig.0001457)
Supplement: S1 Fig — (DOCX) [file pdig.0001457.s002.docx]

## S1 Fig. Supplementary analysis: Forest plot of predictors of non-adherence


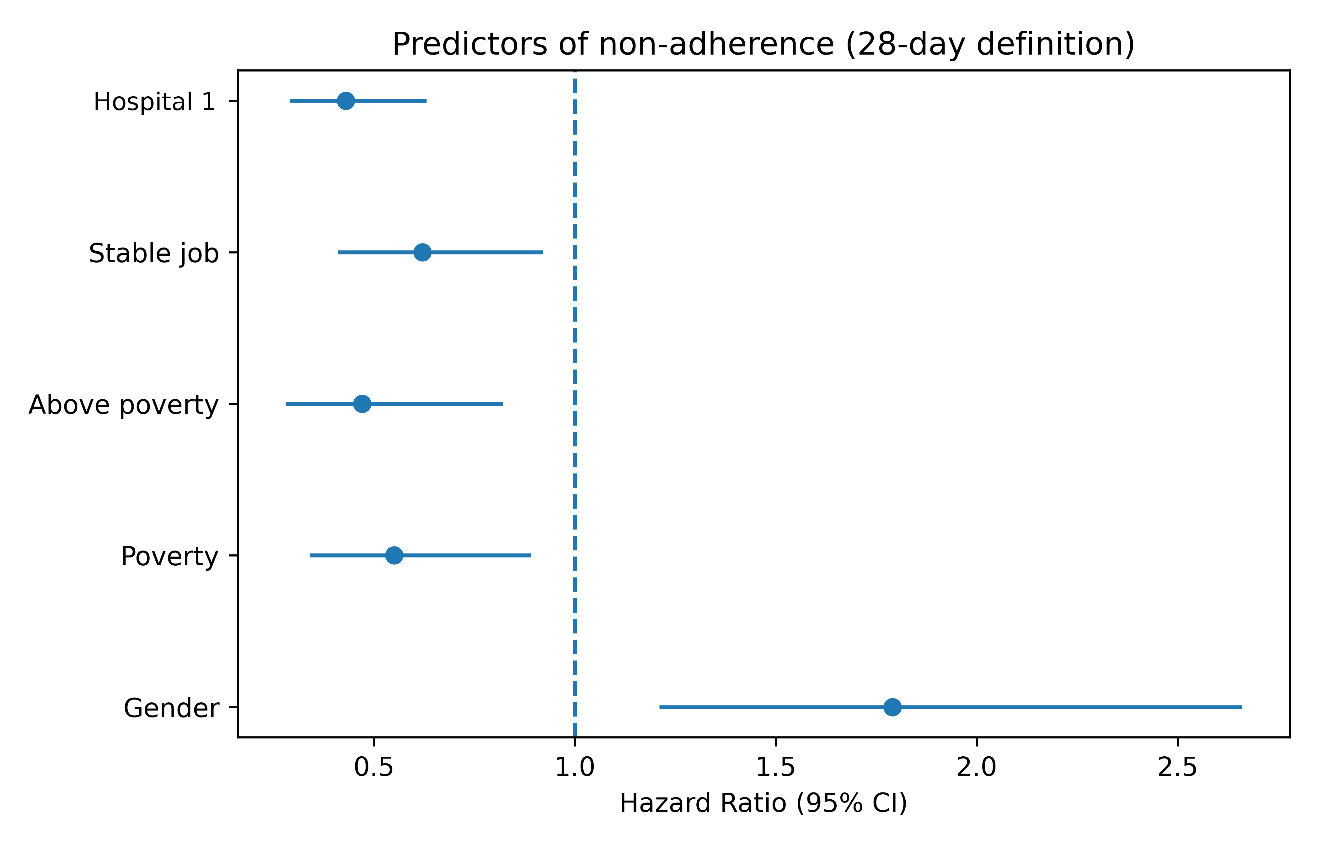


Forest plot showing hazard ratios and 95% confidence intervals for predictors of non-adherence using the 28-day definition.
