# Supplementary material for: User engagement in the tuberculosis treatment support tools intervention and its impact on treatment outcomes: A secondary analysis of a pragmatic trial
Source: PLOS Digit Health. 2026 Jul 2;5(7):e0001457. doi: 10.1371/journal.pdig.0001457 (PMC13327242; doi:10.1371/journal.pdig.0001457)
Supplement: S2 Fig — (DOCX) [file pdig.0001457.s003.docx]

## S2 Fig. Supplementary analysis: Structural equation model of engagement


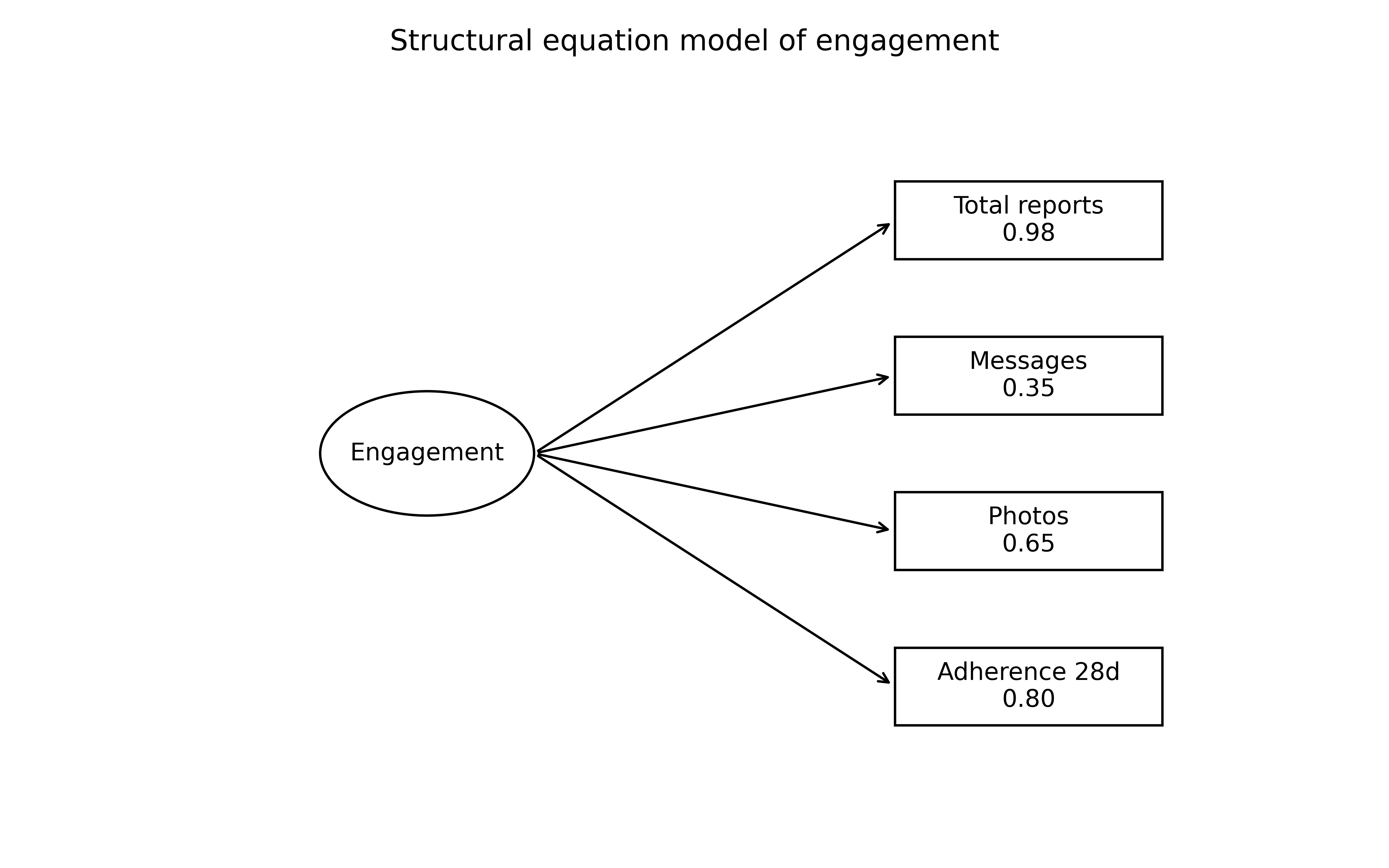


Structural equation model representing the latent engagement construct and standardized factor loadings.
