# Supplementary material for: User engagement in the tuberculosis treatment support tools intervention and its impact on treatment outcomes: A secondary analysis of a pragmatic trial
Source: PLOS Digit Health. 2026 Jul 2;5(7):e0001457. doi: 10.1371/journal.pdig.0001457 (PMC13327242; doi:10.1371/journal.pdig.0001457)
Supplement: S1 Table — (DOCX) [file pdig.0001457.s004.docx]

## S1 Table. Supplementary analysis: Non-adherence according to 14-day and 28-day cut-off definitions (N = 252)

| Outcome definition | Adherent n (%) | Non-adherent n (%) |
| --- | --- | --- |
| 14-day cut-off | 106 (42.1) | 146 (57.9) |
| 28-day cut-off | 130 (51.6) | 122 (48.4) |

*Using the 14-day definition identified 24 additional non-adherent participants compared with the 28-day definition.*
