# Supplementary material for: User engagement in the tuberculosis treatment support tools intervention and its impact on treatment outcomes: A secondary analysis of a pragmatic trial
Source: PLOS Digit Health. 2026 Jul 2;5(7):e0001457. doi: 10.1371/journal.pdig.0001457 (PMC13327242; doi:10.1371/journal.pdig.0001457)
Supplement: S2 Table — (DOCX) [file pdig.0001457.s005.docx]

## S2 Table. Supplementary analysis: Cox proportional hazards models for predictors of non-adherence at 14 and 28 days

| Variable | HR (95% CI) – 28 days | p-value | HR (95% CI) – 14 days | p-value |
| --- | --- | --- | --- | --- |
| Gender | 1.79 (1.21–2.66) | 0.004 | 1.71 (1.19–2.44) | 0.004 |
| *poverty | 0.55 (0.34–0.89) | 0.015 | 0.64 (0.41–0.99) | 0.047 |
| *above poverty | 0.47 (0.28–0.82) | 0.007 | 0.49 (0.30–0.82) | 0.006 |
| Stable employment | 0.62 (0.41–0.92) | 0.018 | 0.58 (0.40–0.83) | 0.003 |
| Hospital 1 (referral) | 0.43 (0.29–0.63) | <0.001 | 0.46 (0.32–0.65) | <0.001 |

*HR: hazard ratio; CI: confidence interval. * Reference category: below poverty line*

*Results are not different using the 14-day or 28-day definition*
