# Supplementary material for: User engagement in the tuberculosis treatment support tools intervention and its impact on treatment outcomes: A secondary analysis of a pragmatic trial
Source: PLOS Digit Health. 2026 Jul 2;5(7):e0001457. doi: 10.1371/journal.pdig.0001457 (PMC13327242; doi:10.1371/journal.pdig.0001457)
Supplement: S3 Table — (DOCX) [file pdig.0001457.s006.docx]

## S4 Table. Supplementary analysis: Goodness-of-fit indices for the structural equation model

| **Fit index** | **Value** | **Interpretation** |
| --- | --- | --- |
| Chi-square model vs saturated, p-value | 0.312 | Adequate fit |
| RMSEA | 0.026 | Excellent fit |
| RMSEA 90% CI | 0.000–0.131 | Acceptable precision |
| p-close | 0.511 | Supports close fit |
| CFI | 0.999 | Excellent fit |
| TLI | 0.997 | Excellent fit |
| SRMR | 0.017 | Excellent fit |
| Coefficient of determination | 0.835 | High explained variance |

RMSEA: Root mean squared error of approximation

p-close: Probability RMSEA <= 0.05

CFI: Comparative fit index

TLI: Tucker–Lewis index

SRMR: Standardized root mean squared residual
