# Supplementary material for: User engagement in the tuberculosis treatment support tools intervention and its impact on treatment outcomes: A secondary analysis of a pragmatic trial
Source: PLOS Digit Health. 2026 Jul 2;5(7):e0001457. doi: 10.1371/journal.pdig.0001457 (PMC13327242; doi:10.1371/journal.pdig.0001457)
Supplement: Attachment — Submitted filename: PDIG-D-25-00218_rev1.pdf [file pdig.0001457.s007.pdf]

PLOS Digital Health

User Engagement in the Tuberculosis Treatment Support Tools Intervention and Its Impact on Treatment Outcomes: A Secondary Analysis of a Pragmatic Trial

--Manuscript Draft--

Manuscript Number:	PDIG-D-25-00218
Article Type:	Research Article
Full Title:	User Engagement in the Tuberculosis Treatment Support Tools Intervention and Its Impact on Treatment Outcomes: A Secondary Analysis of a Pragmatic Trial
Short Title:	User Engagement and Treatment Outcomes in TB-TST
Corresponding Author:	Sarah J Iribarren, Ph.D.<br>University of Washington<br>Seattle, WA UNITED STATES OF AMERICA
Order of Authors:	Sarah J Iribarren, Ph.D.
	Jason Rupp
	Jennifer Sprecher
	Barry Lutz
	Fernando Rubinstein
Keywords:	tuberculosis; digital health; secondary analysis; pragmatic trial
Abstract:	Background: The effectiveness of digital adherence technologies (DATs) in improving health behaviors depends on user engagement, yet the relationship between engagement patterns, user characteristics, and treatment outcomes remains underexplored. The TB Treatment Support Tools (TB-TST) is a DAT featuring a patient-centered app that connects users to treatment supporters and incorporates drug metabolite tests to confirm adherence. This study evaluates engagement with the Companion app in a pragmatic randomized controlled trial, identifying factors associated with adherence and treatment outcomes. Methods and Findings: Engagement data were captured app interactions (n=277) during a 180-day TB treatment period. Adherence was assessed via daily self-reports of medication intake and weekly drug metabolite test photo submissions. Participants could also report side effects, request assistance, or message treatment supporters. Survival analysis identified characteristics associated with non-adherence (28 days without reporting), while logistic regression examined associations between adherence and app engagement with treatment outcome. A latent variable for engagement was developed using confirmatory factor analysis (CFA), incorporating metrics such as medication reports, photo submissions, side effects, message exchange, and overall adherence. Participants submitted 24,902 medication reports, 2,926 messages, 2,465 photos, 1235 side effect reports, and 128 help requests. Adherence declined over time, with 78% adherent at 60 days and 50% at 180 days. Non-adherence was more common among males, those living at or below poverty, individuals without stable employment, and certain hospitals. Non-adherence was linked to lower odds of success (OR 0.48 [0.22 - 1.03]). Higher engagement standardized scores, derived from CFA, were associated with 2.2 times higher odds of success per standard deviation increase. Conclusions: User engagement with the Companion app features significantly influenced TB treatment outcomes. Strategies to sustain engagement, particularly among high-risk groups, are essential to improve adherence and maximize the benefits of DATs like the TB-TSTs.
Additional Information:	
Question	Response
**Government Employee**<br><br>Are you or any of the contributing authors	No - No authors are employees of the U.S. government.

Powered by Editorial Manager® and ProduXion Manager® from Aries Systems Corporation

an employee of the United States government?<br><br>Manuscripts authored by one or more US Government employees are not copyrighted, but are licensed under a CC0 Public Domain Dedication, which allows unlimited distribution and reuse of the article for any lawful purpose. This is a legal requirement for US Government employees.<br><br>This will be typeset if the manuscript is accepted for publication.	
**Financial Disclosure**<br><br>Enter a financial disclosure statement that describes the sources of funding for the work included in this submission. Review the submission guidelines for detailed requirements. View published research articles from *PLOS Digital Health* for specific examples.<br><br>This statement is required for submission and **will appear in the published article** if the submission is accepted. Please make sure it is accurate.<br><br>**Funded studies**<br>Enter a statement with the following details:<br>• Initials of the authors who received each award<br>• Grant numbers awarded to each author<br>• The full name of each funder<br>• URL of each funder website<br>• Did the sponsors or funders play any role in the study design, data collection and analysis, decision to publish, or preparation of the manuscript?<br><br>Did you receive funding for this work?	Yes
Please add funding details.<br>as follow-up to "**Financial Disclosure**<br><br>Enter a financial disclosure statement that	This study was funded by the United States National Institute of Health, National Institute of Allergy and Infectious Diseases (R01AI147129: Iribarren, Rubinstein), and is building on the development research funded by the National Institute of Nursing Research (K23NR017210: Iribarren). The content is the sole responsibility of the

describes the sources of funding for the work included in this submission. Review the submission guidelines for detailed requirements. View published research articles from *PLOS Digital Health* for specific examples.<br><br>This statement is required for submission and **will appear in the published article** if the submission is accepted. Please make sure it is accurate.<br><br>**Funded studies**<br>Enter a statement with the following details:<br>• Initials of the authors who received each award<br>• Grant numbers awarded to each author<br>• The full name of each funder<br>• URL of each funder website<br>• Did the sponsors or funders play any role in the study design, data collection and analysis, decision to publish, or preparation of the manuscript?<br><br>Did you receive funding for this work?"	authors and does not represent the official views of the National Institutes of Health. The funders had no role in study design, data collection and analysis, decision to publish, or preparation of the manuscript.
Please select the country of your main research funder (please select carefully as in some cases this is used in fee calculation).<br>as follow-up to "**Financial Disclosure**<br><br>Enter a financial disclosure statement that describes the sources of funding for the work included in this submission. Review the submission guidelines for detailed requirements. View published research articles from *PLOS Digital Health* for specific examples.<br><br>This statement is required for submission and **will appear in the published article** if the submission is accepted. Please make sure it is accurate.	UNITED STATES - US

**Funded studies**<br>Enter a statement with the following details:<br>• Initials of the authors who received each award<br>• Grant numbers awarded to each author<br>• The full name of each funder<br>• URL of each funder website<br>• Did the sponsors or funders play any role in the study design, data collection and analysis, decision to publish, or preparation of the manuscript?<br><br>Did you receive funding for this work?"	
**Competing Interests**<br><br>On behalf of all authors, disclose any competing interests that could be perceived to bias this work.<br><br>This statement will be typeset if the manuscript is accepted for publication.<br><br>Review the instructions link below and PLOS Digital Health's competing interests policy to determine what information must be disclosed at submission.	Authors have no competing interests
**Data Availability**<br><br>Provide a **Data Availability Statement** in the box below. This statement should detail where the data used in this submission can be accessed. This statement will be typeset if the manuscript is accepted for publication.<br><br>Before publication, authors are required to make all data underlying their findings fully available, without restriction. Review our PLOS Data Policy page for detailed information on this policy. Instructions for writing your Data Availability statement can be accessed via the Instructions link below.	The data will be made available as a minimal data set will be submitted or entered into a data repository

Manuscript_format free with tables and figures
Click here to access/download;Manuscript;App Engagement Manuscript_4.2.25.pdf

Title: User Engagement in the Tuberculosis Treatment Support Tools Intervention and Its Impact on Treatment Outcomes: A Secondary Analysis of a Pragmatic Trial

Authors: Sarah J. Iribarren[1*], Jason Rupp[2], Jennifer Sprecher[1], Barry Lutz[2], Fernando Rubinstein[3]

Affiliations:

1. Biobehavioral Nursing and Health Informatics, University of Washington, Seattle, Washington, United States of America
2. Department of Bioengineering, University of Washington, Seattle, Washington, United States of America
3. Institute for Clinical Effectiveness and Health Policy (IECS), Buenos Aires, Argentina

* Corresponding author: Sarah Iribarren; Email: sjiribar@uw.edu

Short title: User Engagement and Treatment Outcomes in TB-TST

ABSTRACT

Background: The effectiveness of digital adherence technologies (DATs) in improving health behaviors depends on user engagement, yet the relationship between engagement patterns, user characteristics, and treatment outcomes remains underexplored. The TB Treatment Support Tools (TB-TST) is a DAT featuring a patient-centered app that connects users to treatment supporters and incorporates drug metabolite tests to confirm adherence. This study evaluates engagement with the Companion app in a pragmatic randomized controlled trial, identifying factors associated with adherence and treatment outcomes.

Methods and Findings: Engagement data were captured app interactions (n=277) during a 180-day TB treatment period. Adherence was assessed via daily self-reports of medication intake and weekly drug metabolite test photo submissions. Participants could also report side effects, request assistance, or message treatment supporters. Survival analysis identified characteristics associated with non-adherence (28 days without reporting), while logistic regression examined associations between adherence and app engagement with treatment outcome. A latent variable for engagement was developed using confirmatory factor analysis (CFA), incorporating metrics such as medication reports, photo submissions, side effects, message exchange, and overall adherence. Participants submitted 24,902 medication reports, 2,926 messages, 2,465 photos, 1235 side effect reports, and 128 help requests. Adherence declined over time, with 78% adherent at 60 days and 50% at 180 days. Non-adherence was more common among males, those living at or below poverty, individuals without stable employment, and certain hospitals. Non-adherence was linked to lower odds of success (OR 0.48 [0.22 - 1.03]). Higher engagement standardized scores, derived from CFA, were associated with 2.2 times higher odds of success per standard deviation increase.

Conclusions: User engagement with the Companion app features significantly influenced TB treatment outcomes. Strategies to sustain engagement, particularly among high-risk groups, are essential to improve adherence and maximize the benefits of DATs like the TB-TSTs.

INTRODUCTION

The expansion of Digital Adherence Technologies (DATs) offers a promising avenue for cost-effective, patient-centered solutions to address the challenges of complex treatment regimens, particularly for tuberculosis (TB).(1) TB remains the leading infectious killer globally, contributing significantly to morbidity and mortality.(2) Its treatment involves prolonged antibiotic regimens with potential side effects, creating substantial barriers to patient adherence. Poor adherence not only compromises individual health but also exacerbates public health concerns by fostering drug-resistant TB strains and increasing disease transmission.(3)

DATs designed for TB management have predominantly focused on video-based monitoring and smart pill bottles, with fewer studies evaluating the role of interactive mobile applications (apps).(4) Mobile apps hold the potential to enhance personalized treatment supervision, empower patients in self-management, and improve patient-provider communication through advanced functionalities like real-time support and monitoring.(5) However, most existing TB apps target healthcare providers (e.g., dose calculations) or offer general TB information, rather than actively engage patients in their care.(6) Few apps incorporate self-tracking features, side effect monitoring, or objective adherence measures like drug metabolite tests.(6),(7) Moreover, none have been tailored specifically for Latinos or Spanish-speaking populations, despite the Americas region having one of the lowest TB treatment success rates globally and a high proportion of Spanish speakers affected by the disease.(8) Existing reviews of TB-related digital interventions report mixed effectiveness, underscoring the need to critically examine factors influencing their impact.(9)

The success of DATs in improving health outcomes largely hinges on user engagement, often measured as frequency of use.(10) Engagement is crucial for overcoming barriers to care, such as time constraints, travel difficulties, costs, and stigma, while easing the burden on healthcare systems. Despite its importance, engagement remains underexplored, with app-based interventions often evaluated using traditional methods like intent-to-treat analysis, which fail to capture the nuanced relationships between engagement patterns and health outcomes.(11) Understanding how users interact with app features and how these interactions impact treatment outcomes is critical for refining digital interventions, identifying areas for improvement, and supporting users who may require additional engagement strategies.

This study aims to enhance the design and effectiveness of digital health interventions by addressing key research gaps related to user engagement and its impact on treatment outcomes. Specifically, it evaluates participant engagement with the TB Treatment Support Tools (TB-TST) intervention – a DAT that features a comprehensive app that connects patients with treatment supporters and incorporates an objective adherence test (Figure 1). Using data from a pragmatic, multi-site clinical trial, the study explores associations between user characteristics, app usage patterns, and treatment outcomes.

The TB-TST intervention was designed to improve TB treatment outcomes in Argentina, a country with historically low TB treatment success rates (44-67%), high rates of lost-to-follow-up, and a high reliance on self-administered treatment practices.(12) By examining how engagement patterns relate to treatment outcomes, this research aims to provide actionable insights to inform the development of more effective DATs, with the goal of enhancing patient engagement, improving adherence, and ultimately supporting TB treatment success, particularly in high-burden, resource-limited settings.

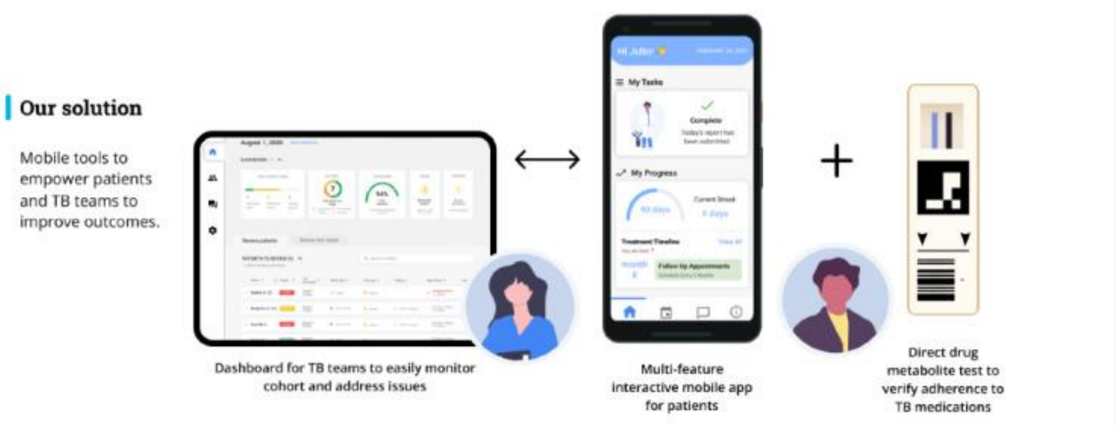

Figure 1. Tuberculosis Treatment Support tools components

METHODS

Overview

This study is a secondary analysis of a parallel-group, pragmatic randomized controlled trial (RCT) that evaluated the efficacy of the TB-TST intervention in improving TB treatment success compared to standard care. This secondary analysis focuses on the intervention arm, examining participant app usage and its association with treatment outcomes. Detailed information on the original trial's study design, procedures, and primary findings are reported elsewhere.(13)

The objectives of this analysis were to: (1) describe app usage patterns, (2) identify participant characteristics associated with adherence to the intervention, and (3) define and analyze the complex concept of engagement, exploring its relationship with treatment outcomes. We hypothesized that higher engagement with the app would be significantly associated with treatment success. To achieve these aims, engagement was operationalized as a multidimensional construct, including metrics of frequency of medication intake reports, photo submissions of drug metabolite urine strip tests, reporting side effects, direct communication with treatment supporters, and overall adherence.

Ethical approval

The primary trial was registered at ClinicalTrials.gov (NCT 04221789), and the Institutional Review Boards of the Ministry of Health of the Province of Buenos Aires (ACTA-2019-15552860-GDEBA-CECMSALGP) and the University of Washington (STUDY00007533) approved the study procedures.

Study Procedures

The data used for these analyses were from baseline, 6-month (180 day) of TB treatment, and final treatment outcomes. Eligibility criteria were (1) newly diagnosed with drug-susceptible TB, (2) aged ≥16 years, and (3) having regular access to a smartphone and able to operate it independently or with assistance. Exclusion criteria included presenting with severe illness requiring hospitalization, confirmed drug resistance, and cohabiting with another participant. Participants were recruited in-person from four large public health hospitals in Argentina on a rolling basis as they were diagnosed with TB from November 2020 through December 2022, 6-months usage completed in July 2023, and final outcomes measures were received in November 2023.

Eligible participants underwent informed consent procedures before enrolling in the study. After enrollment, participants completed an in-person baseline survey and were randomized (in 1:1 ratio) to either control or intervention group. The control group received standard TB care, and the intervention group received standard plus the TB-TST intervention during standard 6-month treatment course.

During enrollment, participants assigned to the intervention group were assisted by trained study personnel to download the app and create a new user account which required an activation key created in the provider dashboard. Participants received verbal instructions and a demonstration of the app's

features and how to complete a metabolite test. They were assisted in entering a test report and uploading a test photo to verify activation and demonstrate understanding. Additionally, participants were provided with a trifold containing visual and written instructions on the app features and performing the drug metabolite test. The app itself also featured an interactive walkthrough and instructional videos covering its main features.

Intervention

The TB-TST intervention was iteratively developed by researchers with clinical and human-center design expertise in collaboration with TB experts and individuals with TB and grounded in behavior change theory and techniques.(14-16) The TB-TST app includes features of: (1) TB education (e.g., written and video information about TB and its treatment), (2) daily survey to submit reports of medication self-administration, side effects, or request of help, or a one-step option to confirm medication was taken and no issues to report, (3) treatment progress visualization (e.g., reporting streak, days in treatment, upcoming appointments), (4) message channels (allowing direct communication with a treatment supporter or participation in an anonymous group forum with other app users), and (5) test instructions and photo submission available on randomized days. Figure 2 illustrates four of these app features.

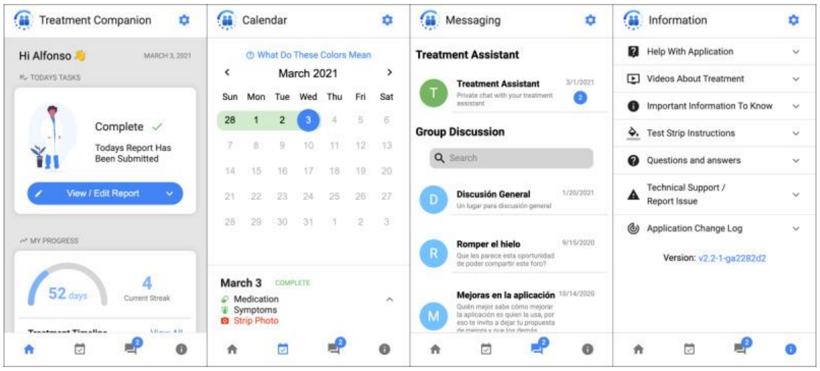

Figure 2. Screenshots of main TB-TST app features

Participants were instructed to report their daily medication intake and, optionally, any potential side effects, questions, or request for assistance through the app for the full course of treatment. Once a week, on a randomly selected day, the app sends a push notification requesting participants to conduct a drug metabolite test and submit a photo of the results. Additionally, participants received weekly push notifications with information, tips, encouragement, behavior change strategies, and reminders tailored to their treatment phase. They also received feedback through the app from treatment supporters, who were health care providers from the recruiting hospitals (four physicians and three social workers). Treatment supporters provided feedback and assistance during usual clinical hours, responding to submissions on the provider dashboard. The dashboard allowed them to review a cohort overview page with summary graphics, communicate with participants, indicate issue resolution, view participant activity history, and view instructions and standard operating procedures.

App data were stored in a database securely hosted on University of Washington infrastructure, managed by the Clinical Informatics Research Group supporting HIPAA compliance, ensuring participant confidentiality, privacy, and data security.

Measures

This study used baseline demographic data, app usage metrics (during the 180-day intervention period), and treatment outcomes obtained from medical records.

Demographics. Baseline demographic variables included age, sex (male, female, nonbinary), education level (primary school, secondary school, some college/post-secondary school), nationality, marital status (married, separated/divorced, single, widowed), current employment status, and household income.

App use. App use metrics included daily reports of medication intake, reports of side effects, test photo submissions, requests for help, and the number of messages sent to or received from treatment supporters.

Adherence to daily medication reporting. Adherence was defined as the number of consecutive days participants reported their medication intake. Non-adherence was operationalized as 28 or more consecutive days of non-reporting (or 14 or more consecutive days in sensitivity analyses). Although treatment duration may have extended beyond 180 days for some participants, adherence was standardized to the 180-day intervention period for consistency.

Engagement. Engagement was conceptualized as a latent variable reflecting the overall pattern of app use. Variables contributing to engagement included the frequency of daily reports of medication intake, side effects, test photo submissions, message activity, and overall adherence. Factor analysis was conducted to confirm whether these interrelated variables collectively explained the latent concept of engagement (see analysis section for details).

Treatment outcome. Treatment outcomes were classified according to WHO definitions: treatment success (cured or completed treatment as recommended), died, lost to follow-up, or not evaluated.(17) Patients who deviated from the study protocol (e.g., diagnosed with drug-resistant TB post-enrollment, extended hospitalization) were assessed to determine whether they were lost to follow-up before the date of protocol deviation.

Analysis

Data processing and statistical analysis were conducted using Python 3.9.12, STATA 18 and R v 4.3.3. Participants were included in the analysis only if they submitted at least one report confirming entry into the system. To ensure data quality, the first submission was excluded, as the protocol required participants to perform a test submission upon enrollment.

Descriptive statistics, including means (SD) for continuous variables and frequencies with percentages for categorical variables, were calculated for baseline variables such as demographics and app use patterns. Chi-square tests were used to assess differences in adherence to daily reporting by socio-demographic characteristics. The dataset used for demographic analysis contained no missing or invalid data.

Survival analysis was applied to evaluate non-adherence similar to Edney et al. 2019.(18) Kaplan-Meier survival curves were constructed to visualize time-to-event data, with log-rank tests used to assess differences between groups (e.g., by sex and other categorical variables). To further explore factors influencing non-adherence, Cox regression and Fine and Gray models were performed. Covariates included participant characteristics such as age, sex, education, employment status, and recruitment site. The time variable was defined as the number of days the app was used, focusing on daily self-report of medication intake. Observations were censored if participants were still using the app at the end of the 180-day intervention or if they were lost-to-follow-up, with loss to follow-up treated as a competing event.

Confirmatory factor analysis (CFA) was conducted to assess the latent variables contributing to *app engagement*. A standardized factor-based score (mean = 0, SD = 1) was generated, where a one-unit change represents one-standard-deviation (1 SD) increase in engagement. This standardized score was incorporated into logistic and Cox regression models to evaluate its association with treatment outcomes (e.g., treatment success, loss to follow-up). Thus, exponentiating the beta coefficients from the logistic regression model reflects the expected change in the odds of the outcomes per 1 SD increase in the engagement score.

RESULTS

Participant Demographics

Participant demographics, stratified by adherence to daily app reporting, are presented in Table 1. The sample was nearly balanced by gender (female 144, 52%), with a mean age of 33.7 years (SD 13.61; range 16-79). Most participants reported completing at least primary school education (261, 94%). Approximately half of the participants were employed at the initiation of treatment (143, 51.6%), fewer than half (119, 42.9%) reported having a spouse or partner. Most participants were Argentinean (213, 76.9%), and nearly half reported a household income at or below the poverty line (127,45.8%).

We assessed potential differences in participant demographics based on adherence to daily app reporting. No significant differences were observed between adherent and nonadherent groups across socio-demographic characteristics.

Table 1: Participant sociodemographic characteristics by adherence to daily reporting

		**Adherent**	**Nonadherent**	***p***
n		130	122	
Gender, n (%)	Female<br>Male	75 (58.1)<br>54 (41.9)	59 (48.5)<br>63 (51.6)	NS
Age, mean (SD)		32.6 (11.5)	33.8 (14.8)	NS
Level of Education, n (%)	Primary<br>Secondary<br>Post-Secondary	25 (19.2)<br>77 (59.2)<br>28 (21.5)	31 (25.6)<br>75 (62)<br>15 (12.4)	NS
Steady Employment, n (%)	Yes	76 (58.5)	55 (45.1)	0.034
Income Level, n (%)	Poverty<br>Above Poverty<br>No Answer	80 (66.1)<br>41 (34)<br>9 (7)	79 (72.5)<br>30 (27.5)<br>13 (10.0)	NS
Marital Status, n (%)	Married / Stable Relationship	71 (54.6)	68 (55.7)	NS
Argentine National, n (%)	Yes	100 (76.9)	93 (76.2)	NS

Participants submitted 23,103 reports of medication intake, averaging 91.7 reports per participant (SD 65.6; range 1-180), 2,926 messages, averaging 11.5 per participant (SD 19.9; range 0-147), 2,343 photo submissions averaging 9.3 per participant (SD 11.4; range 0-66), 1235 reports of side effects averaging 4.9 per participant (SD 13; range 0-87), and 128 requests for help averaging 0.5 per participant (SD 2.1; range 0-27).

Adherence Patterns

Adherence to daily medication reporting varied among participants, as illustrated in the heatmap (Figure 3). Participants exhibited diverse reporting patterns, including consistent daily reporting, intermittent use, early discontinuation, and minimal or no app engagement.

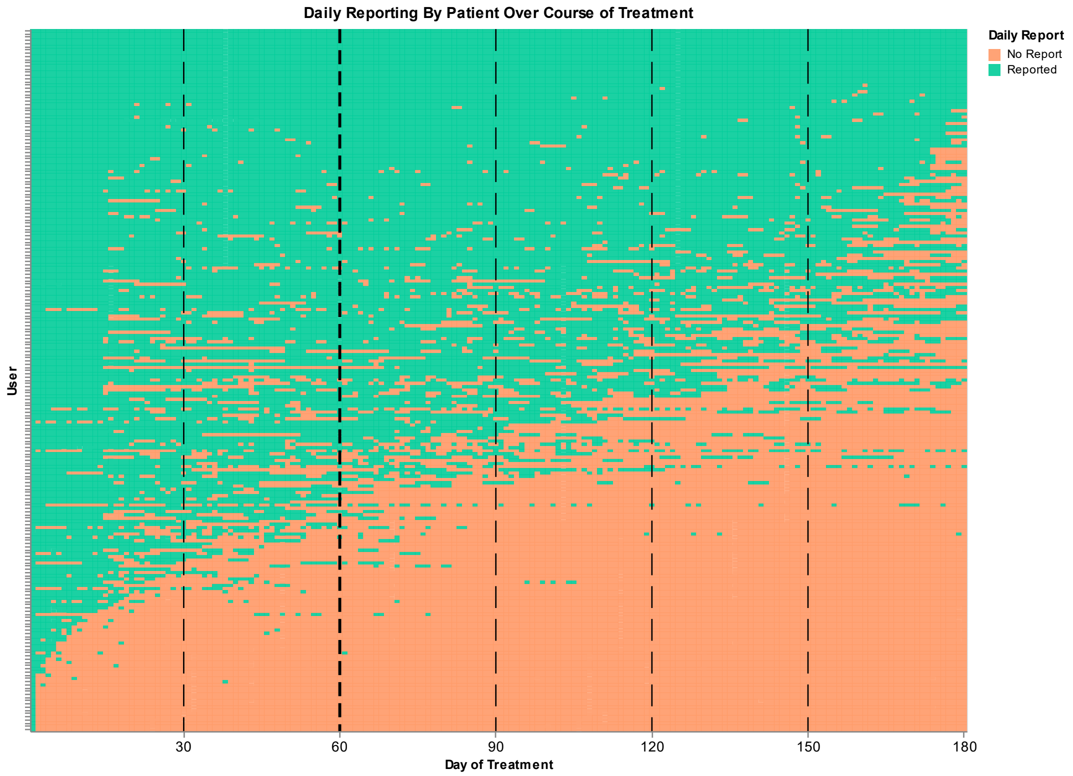

Figure 3. Heatmap of adherence patterns

Side effects reported

Of the 1235 side effect reports, the three most common issues were gastrointestinal-related: abdominal pain, nausea, and appetite loss (Figure 4). Other frequently reported side effects included rash, fever, difficulty breathing, blurry vision, and hives.

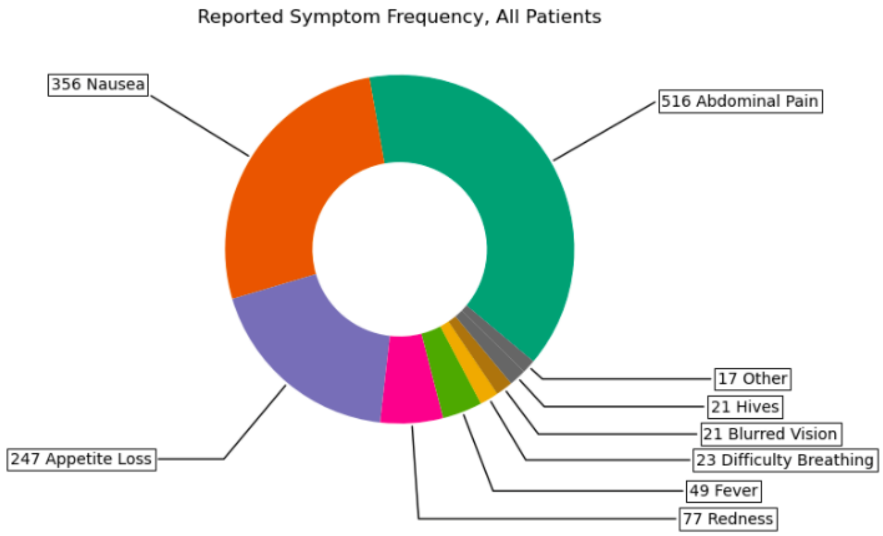

Figure 4. Reported Side effects

Treatment outcomes

Of the 277 participants randomized to the intervention, 234 were included in the final outcome analysis. Participants were excluded if they were not registered in the app (15, 5.4%), registered but did not submit at least one report to confirm the successful app download and activation (7, 2.5%), withdrew immediately after enrollment (1, 0.4%), deviated from protocol within the first 30 days (18, 6.5%), or died during the study period (2, 0.7%). Among the 234 participants included, the majority (197, 84.2%) had the outcome of treatment success, while 37 participants (15.8%) experienced poor treatment outcomes (Table 2).

Table 2. Treatment outcomes

**Outcome**	**N=234 (%)**
Completed Treatment	156 (66.7)
Cured	41 (17.5)
Lost to Follow-up	34 (14.5)
Died	2 (0.01)
Failure	1 (0.004)

Adherence on Treatment Outcomes

Logistic regression analysis demonstrated that nonadherence to the intervention was significantly associated with poor outcomes. Nonadherent participants had lower odds of success (OR 0.48, 95% CI: 0.22 - 0.94), and higher odds of lost to follow-up (OR 2.1, 95% CI: 1.03 - 4.7), adjusting for sex, age, education, income, and employment status.

Factors Associated with Nonadherence (Survival Analysis)

Survival analysis identified significant factors associated with nonadherence: being male, having an income at or below the poverty line, and reporting being unemployed (Table 3). Adherence to daily reporting declined over time, with 78% of participants adherent at 60 days, dropping to 50% by 180 days (Figure 5).

Table 3. Cox model: Time to non-adherence

Variable	HR	95% CI	P value
Male gender	1.79	1.2 - 2.6	0.004
Income			
Below poverty	1		
Poverty line	0.54	0.33 - 0.89	0.015
Above poverty	0.47	0.27 - 0.81	0.007
Stable employment	0.61	0.41 - 0.92	0.018
Hospital Vaccarezza	0.42	0.28 - 0.63	<0.001

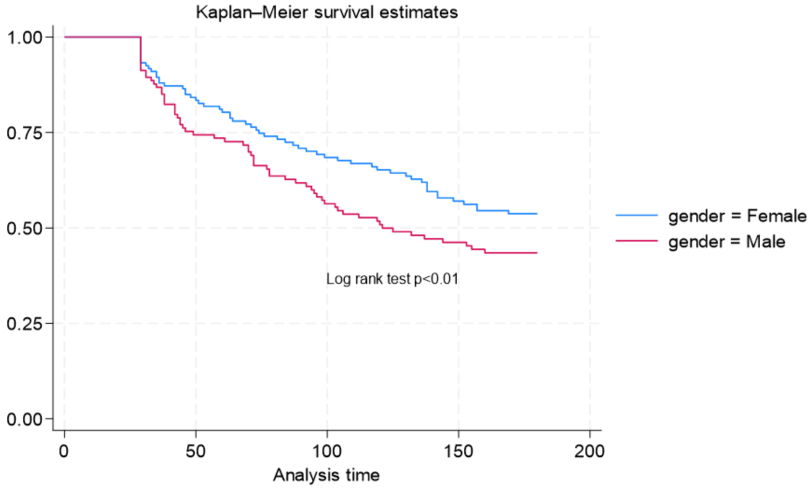

Figure 5. Survival estimates time to nonadherence by gender

Engagement on Treatment Outcome (Confirmatory Factor Analysis)

Confirmatory factor analysis was conducted to identify the underlying construct of app engagement. The variables included in the analysis were daily reports of medication intake, number of messages, number of photo submissions, and adherence. A single factor with an eigenvalue of 2.49 was retained, explaining 62% of the total variance, supporting a unidimensional construct for app engagement. Table 4 presents the factor loadings (pattern matrix) and unique variances for each variable, while Table 5 shows the variable weights used to compute the standardized factor score.

Table 4. Pattern Matrix (Factor Loadings)

Variable (Observed Indicator)	Factor Loading	Unique Variance
Daily medication reports	0.91	0.18
Messages sent/received	0.57	0.68
Photo submissions	0.79	0.37
Adherence	0.85	0.28

Table 5. Weights of app engagement variables

Variable (Observed Indicator)	Weight
Daily medication reports	0.36
Messages sent/received	0.23
Photo submissions	0.32
Adherence	0.34

The standardized engagement score (mean = 0, SD = 1) was entered into logistic regression models to examine its association with treatment outcomes. Adjusting for sex, age, education, income, and employment status, higher engagement was significantly associated with improved outcomes. For each standard deviation increase in engagement score, the odds of treatment success increased (OR 2.2, 95% CI: 1.36-2.53, $p < 0.001$), while the odds of lost to follow-up decreased (OR 0.43, 95% CI: 0.26-0.72, $p < 0.001$).

DISCUSSION

Principal Findings

This study explored the relationship between engagement with a DAT and treatment outcomes among individuals with active TB. Findings demonstrate the potential of our DAT intervention to support treatment adherence and improve outcomes through engagement. Participants who engaged more consistently with the app had significantly higher odds of treatment success, whereas nonadherence to daily reporting was associated with lower success rates and higher loss to follow-up. However, app

usage patterns varied, with a notable decline over time. These results emphasize the importance of understanding both engagement drivers and barriers to inform strategies for sustaining use and refining digital interventions for long and challenging treatment regimens. A novel aspect of this study is the use of factor analysis to conceptualize engagement as a multidimensional construct that incorporates interrelated app features. The approach advances the operationalization of engagement, providing a framework for measuring and improving user interaction with app-based tools in resource-limited, high-burden settings.

App Use Decline and Habit Formation

A key observation was the decline in app usage over time, a trend consistent with previous studies on app-based health interventions.(18, 19) While initial engagement was high, adherence dropped from 78% at 60 days to 50% at 180 days. This decline may reflect the development of behavioral habits, as some participants reported no longer needing reminders after establishing a medication-taking routine. Similarly, participants with minimal side effects or strong social support may have perceived less added value in daily reporting, viewing it as burdensome. To address these patterns, future iterations of the TB-TST intervention could explore phase-based reporting strategies that reduce reporting frequency over time, introduce randomizing reporting on select days, or offer optional interactive features. Such adaptations could sustain engagement while minimizing patient burden during the long treatment course.

Understanding User Engagement

User engagement with digital behavior change interventions is considered important for their effectiveness, therefore, evaluating engagement is important.(20) Confirmatory factor analysis revealed a unidimensional construct of app engagement, combining daily medication reports, messaging, photo

submissions, and adherence. Higher engagement, as captured by this composite score, was significantly associated with better treatment outcomes. This finding highlights the value of factor-based scoring in quantifying engagement, a method that addresses the interdependence of app features and user behavior. Previous research often examines app features separately,(18, 21) neglecting the reality that users may interact with multiple features simultaneously to achieve their health goals. Our approach provides a more holistic understanding of engagement patterns. Importantly, user engagement varied based on individual needs. For some participants, the option to interact directly with a treatment supporter was particularly valuable. However, the success of such features may depend on the quality of interactions, including treatment supporter empathy, responsiveness, and communication style. Ensuring consistency and scalability of personalized support remains a challenge for provider-driven digital interventions.

Challenges with Onboarding and Initial Use

A notable number of participants did not successfully onboard or sustain app use for at least one week. The COVID-19 pandemic exacerbated challenges due to strict travel restrictions and disruptions in clinical follow-up. For those participants without their phones at enrollment, it resulted in difficulty reconnecting to assist them with downloading and onboarding and they fell through the cracks. Barriers to adoption also included male gender, older age, and limited familiarity with technology. These findings align with literature highlighting the difficulty of onboarding older or less tech-savvy users and the challenges of implementing a study during a global pandemic. To address this, efforts should focus on providing targeted onboarding support, follow-up with all after enrollment to assess any technical challenges, particularly to those with limited familiarity with technology, ensure design of user-friendly app interfaces, clearly communicate the app's purpose and benefits, and ensure participants are confident in accessing and navigating the app.

Strengths and Limitations

This study has strengths. First, the large sample size, long data collection period, and large data set enhances the robustness of findings. Second, the development of an app engagement score that accounts for the interrelatedness of multiple app features provides a more comprehensive measure of engagement compared to single-feature metrics. Additionally, the definition of nonadherence (e.g., consecutive missed days) distinguishes between sporadic and prolonged app disengagement, a nuance not captured by overall frequency metrics.

However, there are also some limitations. As a secondary and subgroup analysis of randomized controlled trial data that did not experimentally test the impact of varying levels of engagement on treatment outcomes, causal claims cannot be made. Moreover, self-selection bias likely favored participants with greater technological familiarity, a common limitation in health technology research. Lastly, while the app tracking tool provided general insights into feature usage, its utility for detailed engagement analysis was limited due to data nuances and inconsistencies.

Implications for Digital Health Interventions

This study's findings have important implications for the design and implementation of digital health interventions, particularly for diseases requiring lengthy and complex treatment regimens like TB. By identifying key drivers and barriers of engagement, this study provides a foundation for enhancing the effectiveness of DATs or similar tools. To maximize impact, digital interventions should focus on tailored strategies that address user needs and engagement patterns, improved onboarding processes to reduce barriers to initial adoption, phase-based engagement strategies and ensure consistency of interactive support to sustain long-term use while minimizing burden. Such refinements could enhance

scalability and effectiveness of DATs, particularly in resources limited settings where these technologies hold immense potential for improving health outcomes.

CONCLUSION

This study highlights the critical role of app engagement in achieving successful TB treatment outcomes. Findings emphasize the need for refining the TB-TST intervention to address barriers to onboarding and sustain engagement over time. Future research should explore strategies to optimize user engagement, reduce burdens, ensure consistent treatment support, and tailor tools to diverse patient populations. By addressing these challenges, DATs can more effectively support treatment adherence and improve health outcomes in resource-limited and high-burden settings.

REFERENCES

1. Ridho A, Alfian SD, van Boven JFM, Levita J, Yalcin EA, Le L, et al. Digital Health Technologies to Improve Medication Adherence and Treatment Outcomes in Patients With Tuberculosis: Systematic Review of Randomized Controlled Trials. J Med Internet Res. 2022;24(2):e33062.

2. World Health Organization. Global tuberculosis report 2023. Geneva; 2023. Contract No.: Licence: CC BY-NC-SA 3.0 IGO.

3. Tola HH, Tol A, Shojaeizadeh D, Garmaroudi G. Tuberculosis Treatment Non-Adherence and Lost to Follow Up among TB Patients with or without HIV in Developing Countries: A Systematic Review. Iran J Public Health. 2015;44(1):1-11.

4. Subbaraman R, de Mondesert L, Musiimenta A, Pai M, Mayer KH, Thomas BE, et al. Digital adherence technologies for the management of tuberculosis therapy: mapping the landscape and research priorities. BMJ Glob Health. 2018;3(5):e001018.

5. Triantafyllidis A, Kondylakis H, Votis K, Tzovaras D, Maglaveras N, Rahimi K. Features, outcomes, and challenges in mobile health interventions for patients living with chronic diseases: A review of systematic reviews. Int J Med Inform. 2019;132:103984.

6. Iribarren SJ, Schnall R, Stone PW, Carballo-Dieguez A. Smartphone Applications to Support Tuberculosis Prevention and Treatment: Review and Evaluation. JMIR Mhealth Uhealth. 2016;4(2):e25.

7. Keutzer L, Wicha SG, Simonsson US. Mobile Health Apps for Improvement of Tuberculosis Treatment: Descriptive Review. JMIR Mhealth Uhealth. 2020;8(4):e17246.

8. Tuberculosis en las Ame´ ricas. Informe regional 2021. . Washington, DC: Organizacio´ n Panamericana de la Salud; 2022.

9. Zary M, Mohamed MS, Kafie C, Chilala CI, Bahukudumbi S, Foster N, et al. The performance of digital technologies for measuring tuberculosis medication adherence: a systematic review. BMJ Glob Health. 2024;9(7).

10. Yardley L, Spring BJ, Riper H, Morrison LG, Crane DH, Curtis K, et al. Understanding and Promoting Effective Engagement With Digital Behavior Change Interventions. Am J Prev Med. 2016;51(5):833-42.

11. O'Brien HL, Morton E, Kampen A, Barnes SJ, Michalak EE. Beyond clicks and downloads: a call for a more comprehensive approach to measuring mobile-health app engagement. BJPsych Open. 2020;6(5):e86.

12. Klein K, Bernachea MP, Iribarren S, Gibbons L, Chirico C, Rubinstein F. Correction: Evaluation of a social protection policy on tuberculosis treatment outcomes: A prospective cohort study. PLoS Med. 2019;16(5):e1002826.

13. Iribarren S, Milligan H, Goodwin K, Aguilar Vidrio OA, Chirico C, Telles H, et al. Mobile Tuberculosis Treatment Support Tools (TB-TSTs) to Increase Treatment Success in Tuberculosis Patients in Argentina: Protocol for a Randomized Controlled Trial. JMIR Research Protocols. 2021;Jun 21(10(6):e28094).

14. Iribarren S. Development and Evaluation of an Interactive Mobile Health Intervention to Support Patients with Active Tuberculosis. https://clinicaltrials.gov/ct2/show/NCT03544476?term=Iribarren&draw=2&rank=1: US National Institute of Nursing Research, National Institute of Health; 2018.

15. Iribarren SJ, Rodriguez Y, Lin L, Chirico C, Discacciati V, Schnall R, et al. Converting and expanding a mobile support intervention: Focus group and field-testing findings from individuals in active tuberculosis treatment. Int J Med Inform. 2020;136:104057.

16. Iribarren S, Milligan H, Chirico C, Goodwin K, Schnall R, Telles H, et al. Patient-centered mobile tuberculosis treatment support tools (TB-TSTs) to improve treatment adherence: A pilot randomized controlled trial exploring feasibility, acceptability and refinement needs. The Lancet Regional Health - Americas. 2022;13:100291.

17. World Health Organization. WHO consolidated guidelines on tuberculosis. Module 4: treatment. tuberculosis care and support 2022 [Available from: apps.who.int/iris/handle/10665/3533992022. [LICENSE: CC BY-NC-SA 3.0 IGO].

18. Edney S, Ryan JC, Olds T, Monroe C, Fraysse F, Vandelanotte C, et al. User Engagement and Attrition in an App-Based Physical Activity Intervention: Secondary Analysis of a Randomized Controlled Trial. J Med Internet Res. 2019;21(11):e14645.

19. Moshe I, Terhorst Y, Paganini S, Schlicker S, Pulkki-Råback L, Baumeister H, et al. Predictors of Dropout in a Digital Intervention for the Prevention and Treatment of Depression in Patients With Chronic Back Pain: Secondary Analysis of Two Randomized Controlled Trials. J Med Internet Res. 2022;24(8):e38261.

20. Perski O, Blandford A, West R, Michie S. Conceptualising engagement with digital behaviour change interventions: a systematic review using principles from critical interpretive synthesis. Transl Behav Med. 2017;7(2):254-67.

21. Nardi W, Roy A, Dunsiger S, Brewer J. Analyzing the Impact of Mobile App Engagement on Mental Health Outcomes: Secondary Analysis of the Unwinding Anxiety Program. J Med Internet Res. 2022;24(8):e33696.
