# Supplementary material for: User engagement in the tuberculosis treatment support tools intervention and its impact on treatment outcomes: A secondary analysis of a pragmatic trial
Source: PLOS Digit Health. 2026 Jul 2;5(7):e0001457. doi: 10.1371/journal.pdig.0001457 (PMC13327242; doi:10.1371/journal.pdig.0001457)
Supplement: Attachment — Submitted filename: Reviewer comments_em.pdf [file pdig.0001457.s010.pdf]

- 2,926 messages, 2,465 photos, 1235 side effect reports - add comma to make it 1,235
- Line 54 - start sentence with long form of TB - Tuberculosis; generally recommended formatting for the beginning of sentences. Short form can be used in the middle subsequently
- Lines 110, 112 - start with long form of CFA
- 137-140 - rephrase for better flow. As it is now, it feels rushed. Proposed: Participants were recruited in person from four large public hospitals in Argentina on a rolling basis at the time of TB diagnosis, between November 2020 and December 2022. The 6-month intervention period was completed in July 2023, and final outcome measures were collected in November 2023.

- 228-230 - Propose changing from: Since the score is standardized, the coefficient 229 represents the expected difference in outcome associated with a unit change (one SD) in the engagement 230 score - to "Since the score is standardized, the coefficient represents the expected difference in outcome associated with a one standard deviation increase in the engagement score." - avoids this (one SD)

- Results vs abstract difference in submitted reports: 23,103 vs 24,902 - also photos 2,345 vs 2,465 : Standardize these:

    - Participants submitted 23,103 reports of medication intake, averaging 91.7 reports per participant (SD 244 65.6; range 1-180), 2,926 messages, averaging 11.5 per participant (SD 19.9; range 0-147), 2,343 photo 245 submissions averaging 9.3 per participant (SD 11.4; range 0-66), 1,235 reports of side effects averaging 246 4.9 per participant (SD 13; range 0-87), and 128 requests for help averaging 0.5 per participant (SD 2.1; 247 range 0-27).

    - Update the abstract which reads - Participants submitted 24,902 medication reports, 2,926 messages, 2,465 photos, 1235 side effect reports, and 128 help requests.

- table 1 - line 248
    - non-adherent 122 - 59 and 63 - rounding off artefact to be corrected so that it doesn't add up to more than 100% i.e. 48.5% + 51.6%: cross check all tables.
    - table 2 line 278 - percentages add up to 98.712%

- line 323 - suggest consistency to rule of CFA in full at the beginning of a sentence
- line 394 - Coronavirus disease 2019 (COVID-19)
