# Supplementary material for: User engagement in the tuberculosis treatment support tools intervention and its impact on treatment outcomes: A secondary analysis of a pragmatic trial
Source: PLOS Digit Health. 2026 Jul 2;5(7):e0001457. doi: 10.1371/journal.pdig.0001457 (PMC13327242; doi:10.1371/journal.pdig.0001457)
Supplement: Attachment — Submitted filename: Response to reviewer comments.docx [file pdig.0001457.s009.docx]

**Response to Reviewers**

We sincerely thank the Editor-in-Chief and the reviewers for their thoughtful feedback and valuable suggestions as well as the opportunity to revise and resubmit our manuscript. We believe the revisions have substantially improved the clarity and strength of the manuscript. We welcome any additional feedback and provide below a detailed, point-by-point response to each of the reviewers’ comments.

Reviewer #1:

General Assessment:
 This manuscript provides a valuable and timely secondary analysis exploring app engagement and TB treatment outcomes. The use of confirmatory factor analysis to derive a latent engagement score is a particular strength. The manuscript is clear and well-organized, with relevant implications for digital health design in resource-limited settings.

**Response**: Thank you

Major Comments:
 1. Clarify which components of this secondary analysis were pre-specified in the original trial protocol and which are exploratory.

**Response**: Analysis of level of engagement was planned a priori in the original trial protocol. An additional clarification of the use of confirmatory factor analysis (CFA) has been added to the methods section. Thank you.

2. Expand on the rationale and model selection criteria for using CFA to derive the engagement construct.

**Responses**: We added rational for model selection and criteria for using CFA to derive the engagement construct in the analysis section of the manuscript. We feel this adds clarity to our process and provides context for those who may be less familiar with this methodology.

3. Provide quantification of onboarding issues, such as the number excluded due to failed app registration

**Responses**: We report the number excluded from the main treatment outcome analysis in the results section: Participants were excluded if they were not registered in the app (15, 5.4%), registered but did not submit at least one report to confirm the successful app download and activation (7, 2.5%), withdrew immediately after enrollment (1, 0.4%), deviated from protocol within the first 30 days (18, 6.5%), or died during the study period (2, 0.7%). We have clarified exclusion criteria in the methods section.

4. Elaborate on outcome classifications, especially the small numbers for failure and death; clarify how missing or unclassified data were handled.

**Response**: We added clarification in the methods section that the treatment outcomes were drawn from the medical records and based on the WHO treatment outcome criteria.

5. Discuss possible gender differences in app use patterns and perceived utility, given male sex was associated with nonadherence.

**Response**: Thank you for this suggestion. We have now added a paragraph in the discussion section on gender differences and potential perceived utility given the significant finding of male sex associated with nonadherence.

Minor Comments:
 - In the abstract, consider rephrasing “Engagement data were captured app interactions” to “Engagement data were derived from app interactions” for clarity – optional

**Response**: Thank you for the suggestion. The suggested change has been made.

- Consider visually enhancing Figure 3 with clustering or engagement-level color coding.

**Response**: We have visually enhanced Figure 3 and added lines figure to indicate reporting of 80% or more, 20-80%, and less than 20%. We also removed statement regarding various reporting patterns as the data were not analyzed or separated in that way. The ‘heatmap’ serves as a visual representation of reporting over time and across the cohort.

- Abstract mentions Companion app yet manuscript refers to TB TST-APP - Enhance uniformity for clarity

**Response**: Thank you for bringing this to our attention. We have changed to	be TB-TST app for consistency.

- Specify the data repository or access link in the Data Availability Statement.

**Response**: We have uploaded the de-identified study data to Dryad and have a DOI number to add to the Data Availability Statement. DOI: 10.5061/dryad.x3ffbg7zj.

Suggestions for Future Work:
 - The engagement score could be used in future trials as a stratification variable or mediator in causal pathways.

**Response**: Thank you for this suggestion. We have added this as a potential future	consideration.

 - Including user qualitative feedback could complement the quantitative analysis and provide richer insight into engagement behavior.

**Response**: We conducted qualitative interviews with participants and treatment	supporter which is reported elsewhere. This has been noted in future work.

Summary Recommendation:
This is a well-conducted secondary analysis. With minor revisions highlighted, the manuscript will contribute significantly to digital health and TB care literature.

**Response**: Thank you

**Reviewer #2**:

Line 66: Suggestion to replace ‘Latinos’ with ‘people of Latin American descent’

**Response**: Thank you for this suggestion. This has been changed accordingly.

Line 174: Clarification needed if data on ‘sex’ or ‘gender’ was collected, since ‘sex’ is mentioned here, but ‘gender’ is mentioned in the results

**Response**: Thank you for pointing out this discrepancy. We have changed gender to sex.

Line 176: How was the household income measured? (Monthly/Annual)

**Response**: Household income was captured by asking participants to select their monthly income range from established ranges.

Please report how (if) missing covariate data (e.g., “No Answer” income) were handled; were participants with missing data excluded?

**Response**: In the analysis, income level was not related to treatment outcomes, although it was associated

In Table 2, “Died 2 (0.01)” likely should read “2 (0.9%).”

**Response**: Thank you for identifying this error. It has been corrected.

The trial was conducted during COVID-19, when clinical follow-up was disrupted. If the word limit permits, I suggest you expand on how pandemic-related factors may have influenced engagement and treatment outcomes, and how this context affects applicability to post-pandemic settings.

**Response**: This is an important question that we are currently further exploring through in-depth interviews with participating healthcare systems. In this study, we highlight that the COVID-19 pandemic impacted onboarding and follow-up due to upheaval and challenges faced by the healthcare systems.

Consider including a brief table or appendix summarising common technical difficulties encountered during enrollment (e.g., smartphone ownership, connectivity issues) and any mitigation strategies trial staff employed.

**Response**: Smartphone ownership was an inclusion criteria given that the public healthcare system did not plan to provide smartphones in the future. Connectivity issues were planned for during app development and the app had off-line functionality for participants to be able to submit data when they had connectivity issues and would automatically upload when connection was reestablished. This likely contributed to intermittent reporting patterns. This information has been added to the discussion section. Treatment supporters helped participants with connectivity issues related app access difficulties when they came into clinic.

**Reviewer #3**:

The study “User Engagement in the Tuberculosis Treatment Support Tools Intervention and Its Impact on Treatment Outcomes: A Secondary Analysis of a Pragmatic Trial” aims to describe factors associated with successful use of an app meant to increase adherence to and success with tuberculosis treatment in a population in Argentina. The data was originally collected as part of a larger study, and this analysis focuses on the subgroup who was part of the intervention arm.

The primary findings of this paper are interesting, with figures and tables that are generally easy to follow. The authors clearly identify populations that were less likely to remain adherent to the app, and are able to correlate overall poor adherence to worse success rates with TB treatment. While the methodology here prevents determination of causality, these findings are a useful additional data point to the growing body of literature in this space.

The paper has numerous issues that prevent it from being ready for publication at this time. Notably, the entire text needs to be proofread and edited for readability. There are a significant number of sentences that are run-on, awkwardly worded, or are grammatically odd; this significantly interferes with understanding of the paper. The methods section is quite verbose and contains information that is either not relevant or could be summarized. However, some other details of the methods, such as an explanation regarding the use of drug metabolite test strips, could be expanded (such as how these work or what drug these test for). Likewise, Figure 4 describing the actual side effects experienced by patients seems beyond the scope of what is being discussed here. The discussion section is quite disorganized in its focus and would benefit from a rewrite to more tightly hone in on conclusions that are supported by the data from the study and by literature. There is a robust literature regarding using these types of interventions in patients with HIV and some of these citations may be useful to the authors.

**Response**: Thank you for these comments.

- We have reviewed the entire manuscript and edited throughout to reduce run-on or awkward wording to help with ease of reading.
- The methods section has been revised to reduce sections that were either not relevant or could be summarized. Per Reviewer 1, we have expanded a section on CFA rationale.
- We added a summary of the drug metabolite test strips (how they work and the drug they test for).
- We have removed the figure describing the actual side effects experienced by participants.
- We have revised the discussion section for flow, clarity and reduced run-on, long sentences. We also revised the organization based on study results and the literature.

One of the primary results, regarding non-adherence having a lower odds of success, is listed with a different confidence interval in the abstract than in the results section.

**Response**: Thank you for pointing out this discrepancy. We have corrected.

Overall, the analysis has merit, with findings that would be worth publishing. However, the paper would benefit from substantial proofreading and rewriting several sections to improve the focus.

**Response**: We have revised the manuscript throughout, focusing on streamlining the methods and reworking the discussion section. Thank you for your feedback. We feel this has improved overall flow and readability of the manuscript.
