# Supplementary material for: User engagement in the tuberculosis treatment support tools intervention and its impact on treatment outcomes: A secondary analysis of a pragmatic trial
Source: PLOS Digit Health. 2026 Jul 2;5(7):e0001457. doi: 10.1371/journal.pdig.0001457 (PMC13327242; doi:10.1371/journal.pdig.0001457)
Supplement: Attachment — Submitted filename: Response to Reviewer comments 2.docx [file pdig.0001457.s011.docx]

We sincerely thank the Editor-in-Chief and the reviewers for their thoughtful feedback and valuable suggestions on our resubmission as well as the opportunity to revise and resubmit our manuscript. We believe the additional revisions have improved the clarity and strength of the manuscript. We welcome any additional feedback and provide below a detailed, point-by-point response to each of the reviewers’ comments in blue.

**Reviewer #1**:

Line 184 - 180 days, and not 180-days
Line 204 - "including" instead of "included"

These edits have been made. Thank you.

Line 228 - no differences detected between adherent and non adherent. How about table 1 - variable, "steady employment"? was p value set at <0.05 or <0.01?

Thanks for the observation. Steady employment was the only significant difference. We will correct the sentence highlighting this difference and added that significance was set at <0.01.

Line 243 - grammar - users "who" submitted and not "which" submitted

These edits have been made. Thank you.

Table 251 - the totals do not add up to n=275, those not evaluated should be 43 and not 41 - counting from information in lines 252-258, if not mistaken - please clarify

This is correct, those not evaluated were 43 out of the 277 assigned to the intervention. We have now added a CONSORT-like analysis flow diagram for clarity and have made corrections.

Clarify on the variable - Hospital Vaccarezza - which is a TB referral facility? the reader not familiar with local health systems will find this difficult to interpret

Thank you for pointing this out. We agree. The hospital name was edited to say TB Referral Hospital 1.

Lines 364-365 - suggested rephrasing for accuracy - Our findings highlight the need to tailor digital health tools to users’ preferences, routines, and engagement patterns, particularly for conditions requiring long-term treatment, such as TB.

Thank you for this suggestion. This sentence has been changed to the reviewer’s recommendation.

line 370 - plural - interventions, instead of intervention

This edit has been made. Thank you

**Reviewer #2**:

(No Response)

**Reviewer #4**:

1. Does this manuscript meet PLOS Digital Health’s publication criteria? Yes, it is methodologically and ethically rigorous, technically sound, and conclusions are supported by the data.
Thank you. No changes made.

2. Has the statistical analysis been performed appropriately and rigorously? Yes, analyses are appropriate and rigorous, but some clarifications and consistency checks are required.
Thank you. No changes made.

3. Have the authors made all data underlying the findings fully available? No, the Data Availability Statement is incomplete and must specify repository, DOI, and exact data/code provided.

The following was added to the end of the manuscript, and the link has now been published and should be accessible:

**Data Availability Statement**

After de-identification, individual trial data has been made openly and publicly available and can be found in:   User engagement in the tuberculosis treatment support tools intervention and its impact on treatment outcomes: A secondary analysis of a pragmatic trial

DRYAD: DOI: 10.5061/dryad.x3ffbg7zj;

4. Is the manuscript presented in an intelligible fashion and written in standard English? Yes, it is clear and in standard English, with only minor formatting and consistency fixes needed.
We have reread the manuscript in full to try to fix formatting issues and inconsistencies. Please see track-change version for careful reading and editing for formatting and consistency fixes. Thank you.

Major Revisions Required

1. Data Availability: Deposit the full de-identified dataset, data dictionary, and analysis code in a public repository; update the Data Availability Statement with repository name and DOI/URL.
 Statement has been added to manuscript including these details. Thank you.

The following was added to the manuscript, and the link has now been published and should be accessible:

**Data Availability Statement**

After de-identification, individual trial data has been made openly and publicly available and can be found in:   User engagement in the tuberculosis treatment support tools intervention and its impact on treatment outcomes: A secondary analysis of a pragmatic trial. DRYAD: DOI: 10.5061/dryad.x3ffbg7zj;

2. Statistical Consistency: Harmonize odds ratio confidence intervals (Abstract vs Results) and standardize reporting of p-values, percentages, and denominators across tables/figures.
We have harmonized the odds ratio confidence intervals in the Abstract and Results. The OR in the abstract (OR 0.48 [0.22 - 1.03]) has been corrected to match the results report (OR 0.48, 95% CI: 0.22 - 0.94). We have also standardized reporting of p-values, percentages, and denominators across tables/figures.

3. Non-adherence Definition: Clearly define the threshold (28 days vs 14 days) consistently across Methods, Results, and Figures; provide sensitivity analyses in Supplement.

We have defined the 28-day cutoff threshold more clearly in methods and reviewed for consistency across sections.

4. Cox and Competing Risks Models: Confirm proportional hazards assumptions were tested and report results; provide full details and interpretation of Fine-and-Gray models alongside Cox regression.
We added these results to the manuscript. The proportional hazard test based on Schoenfeld residuals shows a non-significant departure from the assumption globally and for each variable in the model (added Table 3). Hazard ratio (HR) from Cox model and sub-hazard ratio (SHR) from Fine and Gray show very similar results (Table 4). Only 11 patients experienced the competing event (default) before the main event (non-adherence at 28 days). These results have been added to results and additional text has been added to the results to explain these findings.

5. Engagement Score: Expand CFA reporting (fit indices, reliability, loadings) and clearly describe computation of the standardized engagement score; consider robustness checks with alternative specifications.
CFA to obtain the standardized factor score is briefly explained on page 10. The Bartlett test of sphericity was highly significant (p<0.001) and the Kaiser–Meyer–Olkin measure of sampling adequacy was > 0.5 (p=0.68). Both conditions are met to warrant the use of a factor model.

A single factor with an eigenvalue of 2.49 was retained, explaining 62% of the total variance, supporting a unidimensional construct for app engagement. The factor loadings are shown in Table 5 and 6 shows the scoring coefficients obtained by the regression scoring method to compute the standardized engagement score as a weighted sum of standardized versions of each variable with weights 0.36, 0.22, 0.32 and 0.34.

To evaluate the goodness of fit of the model, we ran a structural equation model. The Baseline Comparison Indices and the size of Residuals both indicate that the model fits substantially better than the baseline model.

Comparative Fit Index (CFI) = 0.974 and Tucker-Lewis Index (TLI)= 0.921

Standardized Root Mean Squared Residual = 0.045 suggests that the average discrepancy between observed and predicted correlations is small.

The Coefficient of Determination (CD) = 0.957 indicates that the model explains a significant component of the variance

6. Baseline and Outcomes Tables: Provide a full baseline characteristics table with exact p-values or standardized differences; ensure outcome percentages (Table 2) are correctly calculated and consistently expressed.

The exact p-values have been added to baseline characteristics table (Table 1) and the outcome percentages (Table 2) have been corrected. Thank you.

7. Flow Diagram: Add a CONSORT-style participant flow diagram reconciling numbers enrolled, excluded, and analyzed; ensure consistency between text and tables.
We have added a CONSORT-style diagram and excluded the information on reasons for exclusion in the text.

8. Figures: Improve figure clarity (heatmap axis labels, legends, log-rank p-values, color-blind-safe palettes) and ensure adequate resolution for publication.

We have ensured adequate resolution for publication and ran it through PLOS’s NAAS tool and has been fixed. Axis labels have been clarified. This figure represents each day of treatment throughout the 180-day treatment course. There is no analyses requiring log-rank or p-values.

9. Terminology and Formatting: Correct minor grammatical/typographical issues (e.g., “non-adherence” vs “nonadherence”), standardize number formatting, and clarify intervention naming (TB-TST vs TB-TSTs).

These corrections have been made (nonadherence to non-adherence; TB-TSTs to TB-TST). Thank you

10. Intervention and Onboarding Details: Provide more detail on onboarding procedures, fidelity monitoring, and the weekly metabolite test algorithm; expand reporting using the TIDieR checklist.

We have added details consistent with the TIDieR checklist to the intervention description section and Study procedures. See attached TIDieR checklist. Thank you for the suggestion. There was not a metabolite test algorithm. The test was randomly requested, and each test was manually reviewed and coded by a treatment supporter. Fidelity monitoring – monthly reports summarizing were sent to treatment supporters. Treatment supporters reviewed dashboard daily during clinical hours. If patient had issues, they responded.

11. Ethics and Data Protection: Elaborate on participant consent language for digital data reuse, de-identification steps, and safeguards for cross-border data storage.
We added the following to the Ethical approval section: All participants provided written informed consent. Each participant was assigned a unique study identification at screening, and the linkage file was maintained securely within REDCap. For analysis, direct patient identifiers were removed, only study identification numbers were used. Data were stored in approved secure folders accessible only through authenticated, password-protected logins.

12. Limitations and Generalizability: Expand discussion of selection bias, pandemic-related onboarding barriers, site-level differences, and the generalizability of findings beyond the Argentine hospital context.

The limitations and generalizability section has been expanded to discuss the listed concerns. Thank you for highlighting these areas to address.

**Reviewer #5**:

1. The introduction flows better now, but it still repeats background details and could focus more on what makes your study unique.

We have further revised the introduction section to reduce repetition of background details, more clearly emphasize what makes the study unique, and improve phrasing and readability for a more concise flow.

2. The methods section is clearer, though the choice of a 28-day cutoff still needs a stronger explanation.

We appreciate this comment and have clarified our rationale for the 28-day cutoff in the Methods. We selected 28 days a priori to align with monthly TB medication dispensing cycles and standard monthly clinical follow up, which are typical in our study setting. This threshold balances specificity (reducing misclassification due to brief lapses caused by connectivity or short-term illness) with clinical and programmatic relevance (flagging sustained disengagement that would typically prompt outreach or regimen review).

To demonstrate robustness, we conducted sensitivity analyses using a 14day cutoff (reported in the Methods), and we have now added additional threshold checks at 14 days in supplementary analyses; the patterns of results and substantive conclusions were consistent across thresholds. This strengthens confidence that our findings are not an artifact of the chosen cutoff.

3. The description of the drug metabolite test is improved, but adding a note on its reliability would strengthen it.

Results of prior sensitivity and specificity results have been added.

4. The gender analysis is stronger now, but tying it to broader digital health research would give it more context.
The survival analysis was redone to assess gender and age. Based on both models, males have approximately 75-79% higher risk of non-adherence compared to women. Analysis adjusting for age was not a confounder or effect modifier on the relationship between gender and non-adherence.

An additional paragraph at the beginning of the discussion section was added to give more context regarding gender and DAT engagement.

5. COVID-19 is mentioned, but it should be highlighted more clearly as a limitation to generalizability.

The limitations and generalizability section have been expanded to discuss the COVID-19 related issues. Thank you for highlighting these areas to address.

6. The writing has improved, but a final proofread would help smooth out awkward phrasing.

The full draft has been proofread to identify and sooth where we thought could be considered awkward phrasing.

7. PLOS authors have the option to publish the peer review history of their article ([what does this mean?](https://urldefense.com/v3/__https:/track.editorialmanager.com/CL0/https:*2F*2Fjournals.plos.org*2Fdigitalhealth*2Fs*2Feditorial-and-peer-review-process*23loc-peer-review-history/1/010f01990849623b-8cf26345-c590-4300-99f4-7d91d093f9f2-000000/JOgummyNHd0aNHnCC80UcOmBwt4lKmPUKhDFISzQg4c=226__;JSUlJSUl!!K-Hz7m0Vt54!l5UJ1CpQ2Xy_-9GLthBbaAvjQgEGROUNQs4Ecgu8pdd1p4WLI2hbUz_aDA43kjeRRrI35TnfFaA03nE$)). If published, this will include your full peer review and any attached files.


**Do you want your identity to be public for this peer review?** If you choose “no”, your identity will remain anonymous but your review may still be made public.


For information about this choice, including consent withdrawal, please see our [Privacy Policy](https://urldefense.com/v3/__https:/track.editorialmanager.com/CL0/https:*2F*2Fwww.plos.org*2Fprivacy-policy/1/010f01990849623b-8cf26345-c590-4300-99f4-7d91d093f9f2-000000/Pr993y8ooNkL7swBWRXo0DRe28FkjRFzbGvrQb0hG-s=226__;JSUl!!K-Hz7m0Vt54!l5UJ1CpQ2Xy_-9GLthBbaAvjQgEGROUNQs4Ecgu8pdd1p4WLI2hbUz_aDA43kjeRRrI35TnfGPFdMdY$).

Reviewer #1: **Yes:** Eric Mugambi Nturibi

Reviewer #2: **Yes:** Salman Fitrat Khan

Reviewer #4: **Yes:** Sai Nikhitha Malapati

Reviewer #5: **Yes:** Bala Nimmana

[NOTE: If reviewer comments were submitted as an attachment file, they will be attached to this email and accessible via the submission site. Please log into your account, locate the manuscript record, and check for the action link "View Attachments". If this link does not appear, there are no attachment files.]

**Figure resubmission:**

While revising your submission, we strongly recommend that you use PLOS’s NAAS tool ([https://ngplosjournals.pagemajik.ai/artanalysis](https://urldefense.com/v3/__https:/ngplosjournals.pagemajik.ai/artanalysis__;!!K-Hz7m0Vt54!l5UJ1CpQ2Xy_-9GLthBbaAvjQgEGROUNQs4Ecgu8pdd1p4WLI2hbUz_aDA43kjeRRrI35TnfXQKZcbA$)) to test your figure files. NAAS can convert your figure files to the TIFF file type and meet basic requirements (such as print size, resolution), or provide you with a report on issues that do not meet our requirements and that NAAS cannot fix.

After uploading your figures to PLOS’s NAAS tool - [https://ngplosjournals.pagemajik.ai/artanalysis](https://urldefense.com/v3/__https:/ngplosjournals.pagemajik.ai/artanalysis__;!!K-Hz7m0Vt54!l5UJ1CpQ2Xy_-9GLthBbaAvjQgEGROUNQs4Ecgu8pdd1p4WLI2hbUz_aDA43kjeRRrI35TnfXQKZcbA$), NAAS will process the files provided and display the results in the "Uploaded Files" section of the page as the processing is complete. If the uploaded figures meet our requirements (or NAAS is able to fix the files to meet our requirements), the figure will be marked as "fixed" above. If NAAS is unable to fix the files, a red "failed" label will appear above. When NAAS has confirmed that the figure files meet our requirements, please download the file via the download option, and include these NAAS processed figure files when submitting your revised manuscript.

All figures were run through the PLOS’s NAAS tool and have been fixed.

**Reproducibility:**

To enhance the reproducibility of your results, we recommend that authors of applicable studies deposit laboratory protocols in protocols.io, where a protocol can be assigned its own identifier (DOI) such that it can be cited independently in the future. Additionally, PLOS ONE offers an option to publish peer-reviewed clinical study protocols. Read more information on sharing protocols at [https://plos.org/protocols?utm_medium=editorial-email&utm_source=authorletters&utm_campaign=protocols](https://urldefense.com/v3/__https:/plos.org/protocols?utm_medium=editorial-email&utm_source=authorletters&utm_campaign=protocols__;!!K-Hz7m0Vt54!l5UJ1CpQ2Xy_-9GLthBbaAvjQgEGROUNQs4Ecgu8pdd1p4WLI2hbUz_aDA43kjeRRrI35TnfcsJew9Q$)

Study protocol has been published: doi: 10.2196/28094

**Journal Requirements:**

1. Please provide a detailed online Financial Disclosure statement. This is published with the article. It must therefore be completed in full sentences and contain the exact wording you wish to be published.

a) State the initials, alongside each funding source, of each author to receive each grant, if applicable. For example: “This work was supported by the National Institutes of Health (####### to AM; ###### to CJ) and the National Science Foundation (###### to AM).”

This information has been verified

b) State what role the funders took in the study. If the funders had no role in your study, please state: “The funders had no role in study design, data collection and analysis, decision to publish, or preparation of the manuscript.”

For more information, please see our guidelines: [https://journals.plos.org/digitalhealth/s/submission-guidelines#loc-financial-disclosure-statement](https://urldefense.com/v3/__https:/journals.plos.org/digitalhealth/s/submission-guidelines*loc-financial-disclosure-statement__;Iw!!K-Hz7m0Vt54!l5UJ1CpQ2Xy_-9GLthBbaAvjQgEGROUNQs4Ecgu8pdd1p4WLI2hbUz_aDA43kjeRRrI35TnfKZ2Ty1g$)

The funder role is included

2. Please ensure that the funders and grant numbers match between the Financial Disclosure field and the Funding Information tab in your submission form. Note that the funders must be provided in the same order in both places as well.

The funders and grant numbers and order match between the Financial Disclosure field and Funding Information tab.

3. Please update your online Competing Interests statement. If you have no competing interests to declare, please state: “The authors have declared that no competing interests exist.”

This has been updated

4. Thank you for submitting your data to a repository. We have been unable to access the data using the link/accession number you provided. Please contact the repository to ensure that the links and accession numbers are valid. If a link or accession number needs updating, please provide a revised Data Availability Statement including the information.

The link has now been published and should be accessible.

5. Please provide separate main figure files in .tif or .eps format only and ensure that all files are under our size limit of 10MB. You may leave the embedded figures in the manuscript.

For more information about how to convert your figure files please see our guidelines: [https://journals.plos.org/digitalhealth/s/figures](https://urldefense.com/v3/__https:/journals.plos.org/digitalhealth/s/figures__;!!K-Hz7m0Vt54!l5UJ1CpQ2Xy_-9GLthBbaAvjQgEGROUNQs4Ecgu8pdd1p4WLI2hbUz_aDA43kjeRRrI35TnfNIfYy0w$)

Figure 3 was changed to .tif format and is under the size limit of 10MB

6. Please ensure that you refer to Figure 3 in your text as, if accepted, production will need this reference to link the reader to the figure.

This has been corrected. Thank you.

7. If the reviewer comments include a recommendation to cite specific previously published works, please review and evaluate these publications to determine whether they are relevant and should be cited. There is no requirement to cite these works unless the editor has indicated otherwise.

Reviewer's Responses to Questions

**Comments to the Author**

1. If the authors have adequately addressed your comments raised in a previous round of review and you feel that this manuscript is now acceptable for publication, you may indicate that here to bypass the “Comments to the Author” section, enter your conflict of interest statement in the “Confidential to Editor” section, and submit your "Accept" recommendation.

Reviewer #1: (No Response)

Reviewer #2: All comments have been addressed

Reviewer #4: All comments have been addressed

Reviewer #5: All comments have been addressed

2. Does this manuscript meet PLOS Digital Health’s [publication criteria](https://urldefense.com/v3/__https:/track.editorialmanager.com/CL0/https:*2F*2Fjournals.plos.org*2Fdigitalhealth*2Fs*2Fjournal-information*23loc-criteria-for-publication/1/010f01990849623b-8cf26345-c590-4300-99f4-7d91d093f9f2-000000/zLVzv_QpGnfOBps4dAO3JdGmrCARuvq0h1JHWCN2YE8=226__;JSUlJSUl!!K-Hz7m0Vt54!l5UJ1CpQ2Xy_-9GLthBbaAvjQgEGROUNQs4Ecgu8pdd1p4WLI2hbUz_aDA43kjeRRrI35TnfO34LXto$)? Is the manuscript technically sound, and do the data support the conclusions? The manuscript must describe methodologically and ethically rigorous research with conclusions that are appropriately drawn based on the data presented.

Reviewer #1: Yes

Reviewer #2: Yes

Reviewer #4: Yes

Reviewer #5: Yes

3. Has the statistical analysis been performed appropriately and rigorously?

Reviewer #1: Yes

Reviewer #2: Yes

Reviewer #4: Yes

Reviewer #5: Yes

4. Have the authors made all data underlying the findings in their manuscript fully available (please refer to the Data Availability Statement at the start of the manuscript PDF file)?

The [PLOS Data policy](https://urldefense.com/v3/__https:/track.editorialmanager.com/CL0/https:*2F*2Fjournals.plos.org*2Fdigitalhealth*2Fs*2Fdata-availability/1/010f01990849623b-8cf26345-c590-4300-99f4-7d91d093f9f2-000000/BrZe_DE63ksx0z-hikqex0gKY1eIhjV52xS_cwzlaQI=226__;JSUlJSU!!K-Hz7m0Vt54!l5UJ1CpQ2Xy_-9GLthBbaAvjQgEGROUNQs4Ecgu8pdd1p4WLI2hbUz_aDA43kjeRRrI35Tnf1L219E8$) requires authors to make all data underlying the findings described in their manuscript fully available without restriction, with rare exception. The data should be provided as part of the manuscript or its supporting information, or deposited to a public repository. For example, in addition to summary statistics, the data points behind means, medians and variance measures should be available. If there are restrictions on publicly sharing data—e.g. participant privacy or use of data from a third party—those must be specified.

Reviewer #1: Yes

Reviewer #2: Yes

Reviewer #4: No

Reviewer #5: Yes

5. Is the manuscript presented in an intelligible fashion and written in standard English?

PLOS Digital Health does not copyedit accepted manuscripts, so the language in submitted articles must be clear, correct, and unambiguous. Any typographical or grammatical errors should be corrected at revision, so please note any specific errors here.

Reviewer #1: Yes

Reviewer #2: Yes

Reviewer #4: Yes

Reviewer #5: Yes
