# Supplementary material for: User engagement in the tuberculosis treatment support tools intervention and its impact on treatment outcomes: A secondary analysis of a pragmatic trial
Source: PLOS Digit Health. 2026 Jul 2;5(7):e0001457. doi: 10.1371/journal.pdig.0001457 (PMC13327242; doi:10.1371/journal.pdig.0001457)
Supplement: Peer review history

# Author comment: Author response to Decision Letter 0

**DOI:** 10.1371/journal.pdig.0001457.r001

**Transfer Alert**

This paper was transferred from another journal. As a result, its full editorial history (including decision letters, peer reviews and author responses) may not be present.

13 Apr 2025

# Peer review: Decision Letter 0

**DOI:** 10.1371/journal.pdig.0001457.r002

**Authors:** Haleh Ayatollahi

13 May 2025

PDIG-D-25-00218

User Engagement in the Tuberculosis Treatment Support Tools Intervention and Its Impact on Treatment Outcomes: A Secondary Analysis of a Pragmatic Trial

PLOS Digital Health

Dear Dr. Iribarren,

Thank you for submitting your manuscript to PLOS Digital Health. After careful consideration, we feel that it has merit but does not fully meet PLOS Digital Health's publication criteria as it currently stands. Therefore, we invite you to submit a revised version of the manuscript that addresses the points raised during the review process.

Please submit your revised manuscript within 60 days Jul 12 2025 11:59PM. If you will need more time than this to complete your revisions, please reply to this message or contact the journal office at digitalhealth@plos.org. When you're ready to submit your revision, log on to https://www.editorialmanager.com/pdig/ and select the 'Submissions Needing Revision' folder to locate your manuscript file.

Please include the following items when submitting your revised manuscript:

* A rebuttal letter that responds to each point raised by the editor and reviewer(s). You should upload this letter as a separate file labeled 'Response to Reviewers'. This file does not need to include responses to any formatting updates and technical items listed in the 'Journal Requirements' section below.

* A marked-up copy of your manuscript that highlights changes made to the original version. You should upload this as a separate file labeled 'Revised Manuscript with Track Changes'.

* An unmarked version of your revised paper without tracked changes. You should upload this as a separate file labeled 'Manuscript'.

If you would like to make changes to your financial disclosure, competing interests statement, or data availability statement, please make these updates within the submission form at the time of resubmission. Guidelines for resubmitting your figure files are available below the reviewer comments at the end of this letter.

We look forward to receiving your revised manuscript.

Kind regards,

Haleh Ayatollahi

Section Editor

PLOS Digital Health

Haleh Ayatollahi

Section Editor

PLOS Digital Health

Leo Anthony Celi

Editor-in-Chief

PLOS Digital Health

orcid.org/0000-0001-6712-6626

**Journal Requirements:**

1. We ask that a manuscript source file is provided at Revision. Please upload your manuscript file as a .doc, .docx, .rtf or .tex.

**Additional Editor Comments (if provided):**

[Note: HTML markup is below. Please do not edit.]

**Reviewers' Comments:**

Reviewer's Responses to Questions

**Comments to the Author**

1. Does this manuscript meet PLOS Digital Health’s publication criteria? Is the manuscript technically sound, and do the data support the conclusions? The manuscript must describe methodologically and ethically rigorous research with conclusions that are appropriately drawn based on the data presented.

Reviewer #1: Yes

Reviewer #2: Yes

Reviewer #3: Yes

**********

2. Has the statistical analysis been performed appropriately and rigorously?

Reviewer #1: Yes

Reviewer #2: Yes

Reviewer #3: Yes

**********

3. Have the authors made all data underlying the findings in their manuscript fully available (please refer to the Data Availability Statement at the start of the manuscript PDF file)?

The PLOS Data policy requires authors to make all data underlying the findings described in their manuscript fully available without restriction, with rare exception. The data should be provided as part of the manuscript or its supporting information, or deposited to a public repository. For example, in addition to summary statistics, the data points behind means, medians and variance measures should be available. If there are restrictions on publicly sharing data—e.g. participant privacy or use of data from a third party—those must be specified.

Reviewer #1: Yes

Reviewer #2: Yes

Reviewer #3: Yes

**********

4. Is the manuscript presented in an intelligible fashion and written in standard English?

PLOS Digital Health does not copyedit accepted manuscripts, so the language in submitted articles must be clear, correct, and unambiguous. Any typographical or grammatical errors should be corrected at revision, so please note any specific errors here.

Reviewer #1: Yes

Reviewer #2: Yes

Reviewer #3: No

**********

5. Review Comments to the Author

Please use the space provided to explain your answers to the questions above. You may also include additional comments for the author, including concerns about dual publication, research ethics, or publication ethics. (Please upload your review as an attachment if it exceeds 20,000 characters)

Reviewer #1: General Assessment:

This manuscript provides a valuable and timely secondary analysis exploring app engagement and TB treatment outcomes. The use of confirmatory factor analysis to derive a latent engagement score is a particular strength. The manuscript is clear and well-organized, with relevant implications for digital health design in resource-limited settings.

Major Comments:

1. Clarify which components of this secondary analysis were pre-specified in the original trial protocol and which are exploratory.

2. Expand on the rationale and model selection criteria for using CFA to derive the engagement construct.

3. Provide quantification of onboarding issues, such as the number excluded due to failed app registration

4. Elaborate on outcome classifications, especially the small numbers for failure and death; clarify how missing or unclassified data were handled.

5. Discuss possible gender differences in app use patterns and perceived utility, given male sex was associated with nonadherence.

Minor Comments:

- In the abstract, consider rephrasing “Engagement data were captured app interactions” to “Engagement data were derived from app interactions” for clarity - optional

- Consider visually enhancing Figure 3 with clustering or engagement-level color coding.

- Abstract mentions Companion app yet manuscript refers to TB TST-APP - Enhance uniformity for clarity

- Specify the data repository or access link in the Data Availability Statement.

Suggestions for Future Work:

- The engagement score could be used in future trials as a stratification variable or mediator in causal pathways.

- Including user qualitative feedback could complement the quantitative analysis and provide richer insight into engagement behavior.

Summary Recommendation:

This is a well-conducted secondary analysis. With minor revisions highlighted, the manuscript will contribute significantly to digital health and TB care literature.

Reviewer #2: Line 66: Suggestion to replace ‘Latinos’ with ‘people of Latin American descent’

Line 174: Clarification needed if data on ‘sex’ or ‘gender’ was collected, since ‘sex’ is mentioned here, but ‘gender’ is mentioned in the results

Line 176: How was the household income measured? (Monthly/Annual)

Please report how (if) missing covariate data (e.g., “No Answer” income) were handled; were participants with missing data excluded?

In Table 2, “Died 2 (0.01)” likely should read “2 (0.9%).”

The trial was conducted during COVID-19, when clinical follow-up was disrupted. If the word limit permits, I suggest you expand on how pandemic-related factors may have influenced engagement and treatment outcomes, and how this context affects applicability to post-pandemic settings.

Consider including a brief table or appendix summarising common technical difficulties encountered during enrollment (e.g., smartphone ownership, connectivity issues) and any mitigation strategies trial staff employed.

Reviewer #3: The study “User Engagement in the Tuberculosis Treatment Support Tools Intervention and Its Impact on Treatment Outcomes: A Secondary Analysis of a Pragmatic Trial” aims to describe factors associated with successful use of an app meant to increase adherence to and success with tuberculosis treatment in a population in Argentina. The data was originally collected as part of a larger study, and this analysis focuses on the subgroup who was part of the intervention arm.

The primary findings of this paper are interesting, with figures and tables that are generally easy to follow. The authors clearly identify populations that were less likely to remain adherent to the app, and are able to correlate overall poor adherence to worse success rates with TB treatment. While the methodology here prevents determination of causality, these findings are a useful additional data point to the growing body of literature in this space.

The paper has numerous issues that prevent it from being ready for publication at this time. Notably, the entire text needs to be proofread and edited for readability. There are a significant number of sentences that are run-on, awkwardly worded, or are grammatically odd; this significantly interferes with understanding of the paper. The methods section is quite verbose and contains information that is either not relevant or could be summarized. However, some other details of the methods, such as an explanation regarding the use of drug metabolite test strips, could be expanded (such as how these work or what drug these test for). Likewise, Figure 4 describing the actual side effects experienced by patients seems beyond the scope of what is being discussed here. The discussion section is quite disorganized in its focus and would benefit from a rewrite to more tightly hone in on conclusions that are supported by the data from the study and by literature. There is a robust literature regarding using these types of interventions in patients with HIV and some of these citations may be useful to the authors.

One of the primary results, regarding non-adherence having a lower odds of success, is listed with a different confidence interval in the abstract than in the results section.

Overall, the analysis has merit, with findings that would be worth publishing. However, the paper would benefit from substantial proofreading and rewriting several sections to improve the focus.

**********

6. PLOS authors have the option to publish the peer review history of their article (what does this mean?). If published, this will include your full peer review and any attached files.

**Do you want your identity to be public for this peer review?**  If you choose “no”, your identity will remain anonymous but your review may still be made public.

For information about this choice, including consent withdrawal, please see our Privacy Policy.

Reviewer #1: **Yes:**  Eric Mugambi Nturibi

Reviewer #2: **Yes:**  Salman Fitrat Khan

Reviewer #3: No

**********

[NOTE: If reviewer comments were submitted as an attachment file, they will be attached to this email and accessible via the submission site. Please log into your account, locate the manuscript record, and check for the action link "View Attachments". If this link does not appear, there are no attachment files.]

**Figure resubmission:**

While revising your submission, please upload your figure files to the Preflight Analysis and Conversion Engine (PACE) digital diagnostic tool, https://pacev2.apexcovantage.com/. PACE helps ensure that figures meet PLOS requirements. To use PACE, you must first register as a user. Registration is free. Then, login and navigate to the UPLOAD tab, where you will find detailed instructions on how to use the tool. If you encounter any issues or have any questions when using PACE, please email PLOS at figures@plos.org. Please note that Supporting Information files do not need this step. If there are other versions of figure files still present in your submission file inventory at resubmission, please replace them with the PACE-processed versions.

**Reproducibility:**

To enhance the reproducibility of your results, we recommend that authors of applicable studies deposit laboratory protocols in protocols.io, where a protocol can be assigned its own identifier (DOI) such that it can be cited independently in the future. Additionally, PLOS ONE offers an option to publish peer-reviewed clinical study protocols. Read more information on sharing protocols at https://plos.org/protocols?utm_medium=editorial-email&utm_source=authorletters&utm_campaign=protocols

Attachment Submitted filename: PDIG-D-25-00218_rev1.pdf

# Author comment: Author response to Decision Letter 1

**DOI:** 10.1371/journal.pdig.0001457.r003

2 Jul 2025

Attachment Submitted filename: Response to reviewer comments.docx

# Peer review: Decision Letter 1

**DOI:** 10.1371/journal.pdig.0001457.r004

**Authors:** Haleh Ayatollahi, Haleh Ayatollahi

1 Sep 2025

PDIG-D-25-00218R1

User Engagement in the Tuberculosis Treatment Support Tools Intervention and Its Impact on Treatment Outcomes: A Secondary Analysis of a Pragmatic Trial

PLOS Digital Health

Dear Dr. Iribarren,

Thank you for submitting your manuscript to PLOS Digital Health. After careful consideration, we feel that it has merit but does not fully meet PLOS Digital Health's publication criteria as it currently stands. Therefore, we invite you to submit a revised version of the manuscript that addresses the points raised during the review process.

Please submit your revised manuscript within 60 days Oct 31 2025 11:59PM. If you will need more time than this to complete your revisions, please reply to this message or contact the journal office at digitalhealth@plos.org. When you're ready to submit your revision, log on to https://www.editorialmanager.com/pdig/ and select the 'Submissions Needing Revision' folder to locate your manuscript file.

Please include the following items when submitting your revised manuscript:

* A rebuttal letter that responds to each point raised by the editor and reviewer(s). You should upload this letter as a separate file labeled 'Response to Reviewers'. This file does not need to include responses to any formatting updates and technical items listed in the 'Journal Requirements' section below.

* A marked-up copy of your manuscript that highlights changes made to the original version. You should upload this as a separate file labeled 'Revised Manuscript with Track Changes'.

* An unmarked version of your revised paper without tracked changes. You should upload this as a separate file labeled 'Manuscript'.

If you would like to make changes to your financial disclosure, competing interests statement, or data availability statement, please make these updates within the submission form at the time of resubmission. Guidelines for resubmitting your figure files are available below the reviewer comments at the end of this letter.

We look forward to receiving your revised manuscript.

Kind regards,

Haleh Ayatollahi

Section Editor

PLOS Digital Health

Haleh Ayatollahi

Section Editor

PLOS Digital Health

Leo Anthony Celi

Editor-in-Chief

PLOS Digital Health

orcid.org/0000-0001-6712-6626

**Journal Requirements:**

1. Please provide a detailed online Financial Disclosure statement. This is published with the article. It must therefore be completed in full sentences and contain the exact wording you wish to be published.

a) State the initials, alongside each funding source, of each author to receive each grant, if applicable. For example: “This work was supported by the National Institutes of Health (####### to AM; ###### to CJ) and the National Science Foundation (###### to AM).”

b) State what role the funders took in the study. If the funders had no role in your study, please state: “The funders had no role in study design, data collection and analysis, decision to publish, or preparation of the manuscript.”

For more information, please see our guidelines: https://journals.plos.org/digitalhealth/s/submission-guidelines#loc-financial-disclosure-statement

2. Please ensure that the funders and grant numbers match between the Financial Disclosure field and the Funding Information tab in your submission form. Note that the funders must be provided in the same order in both places as well.

3. Please update your online Competing Interests statement. If you have no competing interests to declare, please state: “The authors have declared that no competing interests exist.”

4. Thank you for submitting your data to a repository. We have been unable to access the data using the link/accession number you provided. Please contact the repository to ensure that the links and accession numbers are valid. If a link or accession number needs updating, please provide a revised Data Availability Statement including the information.

5. Please provide separate main figure files in .tif or .eps format only and ensure that all files are under our size limit of 10MB. You may leave the embedded figures in the manuscript.

For more information about how to convert your figure files please see our guidelines: https://journals.plos.org/digitalhealth/s/figures

6. Please ensure that you refer to Figure 3 in your text as, if accepted, production will need this reference to link the reader to the figure.

7. If the reviewer comments include a recommendation to cite specific previously published works, please review and evaluate these publications to determine whether they are relevant and should be cited. There is no requirement to cite these works unless the editor has indicated otherwise.

**Additional Editor Comments (if provided):**

Reviewer #1:

Reviewer #2:

Reviewer #4:

Reviewer #5:

[Note: HTML markup is below. Please do not edit.]

**Reviewers' Comments:**

Reviewer's Responses to Questions

**Comments to the Author**

1. If the authors have adequately addressed your comments raised in a previous round of review and you feel that this manuscript is now acceptable for publication, you may indicate that here to bypass the “Comments to the Author” section, enter your conflict of interest statement in the “Confidential to Editor” section, and submit your "Accept" recommendation.

Reviewer #1: (No Response)

Reviewer #2: All comments have been addressed

Reviewer #4: All comments have been addressed

Reviewer #5: All comments have been addressed

**********

2. Does this manuscript meet PLOS Digital Health’s publication criteria? Is the manuscript technically sound, and do the data support the conclusions? The manuscript must describe methodologically and ethically rigorous research with conclusions that are appropriately drawn based on the data presented.

Reviewer #1: Yes

Reviewer #2: Yes

Reviewer #4: Yes

Reviewer #5: Yes

**********

3. Has the statistical analysis been performed appropriately and rigorously?

Reviewer #1: Yes

Reviewer #2: Yes

Reviewer #4: Yes

Reviewer #5: Yes

**********

4. Have the authors made all data underlying the findings in their manuscript fully available (please refer to the Data Availability Statement at the start of the manuscript PDF file)?

The PLOS Data policy requires authors to make all data underlying the findings described in their manuscript fully available without restriction, with rare exception. The data should be provided as part of the manuscript or its supporting information, or deposited to a public repository. For example, in addition to summary statistics, the data points behind means, medians and variance measures should be available. If there are restrictions on publicly sharing data—e.g. participant privacy or use of data from a third party—those must be specified.

Reviewer #1: Yes

Reviewer #2: Yes

Reviewer #4: No

Reviewer #5: Yes

**********

5. Is the manuscript presented in an intelligible fashion and written in standard English?

PLOS Digital Health does not copyedit accepted manuscripts, so the language in submitted articles must be clear, correct, and unambiguous. Any typographical or grammatical errors should be corrected at revision, so please note any specific errors here.

Reviewer #1: Yes

Reviewer #2: Yes

Reviewer #4: Yes

Reviewer #5: Yes

**********

6. Review Comments to the Author

Please use the space provided to explain your answers to the questions above. You may also include additional comments for the author, including concerns about dual publication, research ethics, or publication ethics. (Please upload your review as an attachment if it exceeds 20,000 characters)

Reviewer #1: Line 184 - 180 days, and not 180-days

Line 204 - "including" instead of "included"

Line 228 - no differences detected between adherent and non adherent. How about table 1 - variable, "steady employment"? was p value set at <0.05 or <0.01?

Line 243 - grammar - users "who" submitted and not "which" submitted

Table 251 - the totals do not add up to n=275, those not evaluated should be 43 and not 41 - counting from information in lines 252-258, if not mistaken - please clarify

Clarify on the variable - Hospital Vaccarezza - which is a TB referral facility? the reader not familiar with local health systems will find this difficult to interpret

Lines 364-365 - suggested rephrasing for accuracy - Our findings highlight the need to tailor digital health tools to users’ preferences, routines, and engagement patterns, particularly for conditions requiring long-term treatment, such as TB.

line 370 - plural - interventions, instead of intervention

Reviewer #2: (No Response)

Reviewer #4: 1. Does this manuscript meet PLOS Digital Health’s publication criteria? Yes, it is methodologically and ethically rigorous, technically sound, and conclusions are supported by the data.

2. Has the statistical analysis been performed appropriately and rigorously? Yes, analyses are appropriate and rigorous, but some clarifications and consistency checks are required.

3. Have the authors made all data underlying the findings fully available? No, the Data Availability Statement is incomplete and must specify repository, DOI, and exact data/code provided.

4. Is the manuscript presented in an intelligible fashion and written in standard English? Yes, it is clear and in standard English, with only minor formatting and consistency fixes needed.

Major Revisions Required

1. Data Availability: Deposit the full de-identified dataset, data dictionary, and analysis code in a public repository; update the Data Availability Statement with repository name and DOI/URL.

2. Statistical Consistency: Harmonize odds ratio confidence intervals (Abstract vs Results) and standardize reporting of p-values, percentages, and denominators across tables/figures.

3. Non-adherence Definition: Clearly define the threshold (28 days vs 14 days) consistently across Methods, Results, and Figures; provide sensitivity analyses in Supplement.

4. Cox and Competing Risks Models: Confirm proportional hazards assumptions were tested and report results; provide full details and interpretation of Fine-and-Gray models alongside Cox regression.

5. Engagement Score: Expand CFA reporting (fit indices, reliability, loadings) and clearly describe computation of the standardized engagement score; consider robustness checks with alternative specifications.

6. Baseline and Outcomes Tables: Provide a full baseline characteristics table with exact p-values or standardized differences; ensure outcome percentages (Table 2) are correctly calculated and consistently expressed.

7. Flow Diagram: Add a CONSORT-style participant flow diagram reconciling numbers enrolled, excluded, and analyzed; ensure consistency between text and tables.

8. Figures: Improve figure clarity (heatmap axis labels, legends, log-rank p-values, color-blind-safe palettes) and ensure adequate resolution for publication.

9. Terminology and Formatting: Correct minor grammatical/typographical issues (e.g., “non-adherence” vs “nonadherence”), standardize number formatting, and clarify intervention naming (TB-TST vs TB-TSTs).

10. Intervention and Onboarding Details: Provide more detail on onboarding procedures, fidelity monitoring, and the weekly metabolite test algorithm; expand reporting using the TIDieR checklist.

11. Ethics and Data Protection: Elaborate on participant consent language for digital data reuse, de-identification steps, and safeguards for cross-border data storage.

12. Limitations and Generalizability:Expand discussion of selection bias, pandemic-related onboarding barriers, site-level differences, and the generalizability of findings beyond the Argentine hospital context.

Reviewer #5: 1. The introduction flows better now, but it still repeats background details and could focus more on what makes your study unique.

2. The methods section is clearer, though the choice of a 28-day cutoff still needs a stronger explanation.

3. The description of the drug metabolite test is improved, but adding a note on its reliability would strengthen it.

4. The gender analysis is stronger now, but tying it to broader digital health research would give it more context.

5. COVID-19 is mentioned, but it should be highlighted more clearly as a limitation to generalizability.

6. The writing has improved, but a final proofread would help smooth out awkward phrasing.

**********

7. PLOS authors have the option to publish the peer review history of their article (what does this mean?). If published, this will include your full peer review and any attached files.

**Do you want your identity to be public for this peer review?**  If you choose “no”, your identity will remain anonymous but your review may still be made public.

For information about this choice, including consent withdrawal, please see our Privacy Policy.

Reviewer #1: **Yes:**  Eric Mugambi Nturibi

Reviewer #2: **Yes:**  Salman Fitrat Khan

Reviewer #4: **Yes:**  Sai Nikhitha Malapati

Reviewer #5: **Yes:**  Bala Nimmana

**********

[NOTE: If reviewer comments were submitted as an attachment file, they will be attached to this email and accessible via the submission site. Please log into your account, locate the manuscript record, and check for the action link "View Attachments". If this link does not appear, there are no attachment files.]

**Figure resubmission:**

While revising your submission, we strongly recommend that you use PLOS’s NAAS tool (https://ngplosjournals.pagemajik.ai/artanalysis) to test your figure files. NAAS can convert your figure files to the TIFF file type and meet basic requirements (such as print size, resolution), or provide you with a report on issues that do not meet our requirements and that NAAS cannot fix.

After uploading your figures to PLOS’s NAAS tool - https://ngplosjournals.pagemajik.ai/artanalysis, NAAS will process the files provided and display the results in the "Uploaded Files" section of the page as the processing is complete. If the uploaded figures meet our requirements (or NAAS is able to fix the files to meet our requirements), the figure will be marked as "fixed" above. If NAAS is unable to fix the files, a red "failed" label will appear above. When NAAS has confirmed that the figure files meet our requirements, please download the file via the download option, and include these NAAS processed figure files when submitting your revised manuscript.

**Reproducibility:**

To enhance the reproducibility of your results, we recommend that authors of applicable studies deposit laboratory protocols in protocols.io, where a protocol can be assigned its own identifier (DOI) such that it can be cited independently in the future. Additionally, PLOS ONE offers an option to publish peer-reviewed clinical study protocols. Read more information on sharing protocols at https://plos.org/protocols?utm_medium=editorial-email&utm_source=authorletters&utm_campaign=protocols

# Author comment: Author response to Decision Letter 2

**DOI:** 10.1371/journal.pdig.0001457.r005

1 Mar 2026

Attachment Submitted filename: Response to Reviewer comments 2.docx

# Peer review: Decision Letter 2

**DOI:** 10.1371/journal.pdig.0001457.r006

**Authors:** Haleh Ayatollahi, Haleh Ayatollahi, Josep Vidal-Alaball

8 May 2026

User Engagement in the Tuberculosis Treatment Support Tools Intervention and Its Impact on Treatment Outcomes: A Secondary Analysis of a Pragmatic Trial

PDIG-D-25-00218R2

Dear Dr. Iribarren,

We are pleased to inform you that your manuscript 'User Engagement in the Tuberculosis Treatment Support Tools Intervention and Its Impact on Treatment Outcomes: A Secondary Analysis of a Pragmatic Trial' has been provisionally accepted for publication in PLOS Digital Health.

Before your manuscript can be formally accepted you will need to complete some formatting changes, which you will receive in a follow-up email from a member of our team.

Please note that your manuscript will not be scheduled for publication until you have made the required changes, so a swift response is appreciated.

IMPORTANT: The editorial review process is now complete. PLOS will only permit corrections to spelling, formatting or significant scientific errors from this point onwards. Requests for major changes, or any which affect the scientific understanding of your work, will cause delays to the publication date of your manuscript.

If your institution or institutions have a press office, please notify them about your upcoming paper to help maximize its impact. If they'll be preparing press materials, please inform our press team as soon as possible -- no later than 48 hours after receiving the formal acceptance. Your manuscript will remain under strict press embargo until 2 pm Eastern Time on the date of publication. For more information, please contact digitalhealth@plos.org.

Thank you again for supporting Open Access publishing; we are looking forward to publishing your work in PLOS Digital Health.

Best regards,

Josep Vidal-Alaball, Ph,D., M.D., MPH

Section Editor

PLOS Digital Health

***********************************************************

**Additional Editor Comments (if provided):**

**Reviewer Comments (if any, and for reference):**

Reviewer's Responses to Questions

**Comments to the Author**

1. If the authors have adequately addressed your comments raised in a previous round of review and you feel that this manuscript is now acceptable for publication, you may indicate that here to bypass the “Comments to the Author” section, enter your conflict of interest statement in the “Confidential to Editor” section, and submit your "Accept" recommendation.

Reviewer #1: All comments have been addressed

Reviewer #4: All comments have been addressed

Reviewer #5: All comments have been addressed

**********

2. Does this manuscript meet PLOS Digital Health’s publication criteria? Is the manuscript technically sound, and do the data support the conclusions? The manuscript must describe methodologically and ethically rigorous research with conclusions that are appropriately drawn based on the data presented.

Reviewer #1: Yes

Reviewer #4: Yes

Reviewer #5: Yes

**********

3. Has the statistical analysis been performed appropriately and rigorously?

Reviewer #1: Yes

Reviewer #4: Yes

Reviewer #5: Yes

**********

4. Have the authors made all data underlying the findings in their manuscript fully available (please refer to the Data Availability Statement at the start of the manuscript PDF file)?

The PLOS Data policy requires authors to make all data underlying the findings described in their manuscript fully available without restriction, with rare exception. The data should be provided as part of the manuscript or its supporting information, or deposited to a public repository. For example, in addition to summary statistics, the data points behind means, medians and variance measures should be available. If there are restrictions on publicly sharing data—e.g. participant privacy or use of data from a third party—those must be specified.

Reviewer #1: Yes

Reviewer #4: Yes

Reviewer #5: Yes

**********

5. Is the manuscript presented in an intelligible fashion and written in standard English?

PLOS Digital Health does not copyedit accepted manuscripts, so the language in submitted articles must be clear, correct, and unambiguous. Any typographical or grammatical errors should be corrected at revision, so please note any specific errors here.

Reviewer #1: Yes

Reviewer #4: Yes

Reviewer #5: Yes

**********

6. Review Comments to the Author

Please use the space provided to explain your answers to the questions above. You may also include additional comments for the author, including concerns about dual publication, research ethics, or publication ethics. (Please upload your review as an attachment if it exceeds 20,000 characters)

Reviewer #1: attached

copy/pasted here:

Please see these comments and make necessary changes:

2,926 messages, 2,465 photos, 1235 side effect reports - add comma to make it 1,235

Line 54 - start sentence with long form of TB - Tuberculosis; generally recommended formatting for the beginning of sentences. Short form can be used in the middle subsequently

Lines 110, 112 - start with long form of CFA

137-140 - rephrase for better flow. As it is now, it feels rushed. Proposed: Participants were recruited in person from four large public hospitals in Argentina on a rolling basis at the time of TB diagnosis, between November 2020 and December 2022. The 6-month intervention period was completed in July 2023, and final outcome measures were collected in November 2023.

228-230 - Propose changing from: Since the score is standardized, the coefficient

229 represents the expected difference in outcome associated with a unit change (one SD) in the engagement 230 score - to "Since the score is standardized, the coefficient represents the expected difference in outcome associated with a one standard deviation increase in the engagement score." - avoids this (one SD)

Results vs abstract difference in submitted reports: 23,103 vs 24,902 - also photos 2,345 vs 2,465 : Standardize these

Participants submitted 23,103 reports of medication intake, averaging 91.7 reports per participant (SD 244 65.6; range 1-180), 2,926 messages, averaging 11.5 per participant (SD 19.9; range 0-147), 2,343 photo 245 submissions averaging 9.3 per participant (SD 11.4; range 0-66), 1,235 reports of side effects averaging 246 4.9 per participant (SD 13; range 0-87), and 128 requests for help averaging 0.5 per participant (SD 2.1; 247 range 0-27).

Update the abstract which reads - Participants submitted 24,902 medication reports, 2,926 messages, 2,465 photos, 1235 side effect reports, and 128 help requests.

table 1 - line 248

non-adherent 122 - 59 and 63 - rounding off artefact to be corrected so that it doesn't add up to more than 100% i.e. 48.5% + 51.6%: cross check all tables. table 2 line 278 - percentages add up to 98.712%

line 323 - suggest consistency to rule of CFA in full at the beginning of a sentence

line 394 - Coronavirus disease 2019 (COVID-19)

Reviewer #4: The manuscript has improved substantially and is now methodologically sound and well-structured.

The statistical analyses are appropriate, clearly described, and consistently reported across sections.

The addition of the Data Availability Statement with repository link and DOI ensures compliance with PLOS data sharing policies.

The inclusion of a CONSORT-style flow diagram improves transparency and participant tracking.

The engagement construct using CFA is now well-defined and supported by appropriate validation metrics.

The description of the intervention and onboarding procedures is sufficiently detailed and aligns with reporting standards.

Ethical considerations, including consent and data protection, are adequately addressed.

The discussion appropriately interprets findings and acknowledges key limitations, including generalizability.

The manuscript is clearly written, logically organized, and free of major language issues.

No concerns regarding dual publication, research ethics, or publication ethics are identified.

Reviewer #5: It was a great move to add the CONSORT-style flow diagram, as it really clears up the confusion regarding participant numbers and exclusions.

Your explanation for the 28-day cutoff makes perfect sense now, especially considering how medication is dispensed in a real-world clinical setting.

It would be a nice touch to briefly mention the 14-day sensitivity analysis results in the text just to prove the findings hold up under different mirrors.

The discussion on gender is much stronger, though a quick sentence explicitly tying your data to the "masculine norms" theory would help bridge that gap.

Since you’ve added the test sensitivity data, a brief word on how easily patients handled the "at-home" testing process would be very helpful for practitioners.

There are still a few minor typos in the tracked changes, like "losst," which a final quick read-through will easily catch.

**********

7. PLOS authors have the option to publish the peer review history of their article (what does this mean?). If published, this will include your full peer review and any attached files.

**Do you want your identity to be public for this peer review?**  If you choose “no”, your identity will remain anonymous but your review may still be made public.

For information about this choice, including consent withdrawal, please see our Privacy Policy.

Reviewer #1: **Yes:**  ERIC MUGAMBI NTURIBI

Reviewer #4: **Yes:**  DR. Sai Nikhitha Malapati

Reviewer #5: **Yes:**  Bala Nimmana

**********

Attachment Submitted filename: Reviewer comments_em.pdf
